# Lima1 mediates the pluripotency control of membrane dynamics and cellular metabolism

**DOI:** 10.1038/s41467-022-28139-5

**Published:** 2022-02-01

**Authors:** Binyamin Duethorn, Fabian Groll, Bettina Rieger, Hannes C. A. Drexler, Heike Brinkmann, Ludmila Kremer, Martin Stehling, Marie-Theres Borowski, Karina Mildner, Dagmar Zeuschner, Magdalena Zernicka-Goetz, Marc P. Stemmler, Karin B. Busch, Juan M. Vaquerizas, Ivan Bedzhov

**Affiliations:** 1 Embryonic Self-Organization research group, Max Planck Institute for Molecular Biomedicine, Röntgenstraße 20, 48149 Münster, Germany; 2 Regulatory Genomics group, Max Planck Institute for Molecular Biomedicine, Röntgenstraße 20, 48149 Münster, Germany; 3 Institut für Integrative Zellbiologie und Physiologie, University of Münster, Schlossplatz 5, 48149 Münster, Germany; 4 Mass Spectrometry Unit, Max Planck Institute for Molecular Biomedicine, Röntgenstraße 20, 48149 Münster, Germany; 5 Transgenic Facility, Max Planck Institute for Molecular Biomedicine, Röntgenstraße 20, 48149 Münster, Germany; 6 Flow Cytometry Unit, Max Planck Institute for Molecular Biomedicine, Röntgenstraße 20, 48149 Münster, Germany; 7 Electron Microscopy Facility, Max Planck Institute for Molecular Biomedicine, Röntgenstraße 20, 48149 Münster, Germany; 8 Mammalian Embryo and Stem Cell Group, Department of Physiology, Development, and Neuroscience, University of Cambridge, Downing Street, Cambridge, CB2 3EG UK; 9 Plasticity and Self-Organization Group, Division of Biology and Biological Engineering, California Institute of Technology (Caltech), Pasadena, CA 91125 USA; 10 Department of Experimental Medicine 1, Nikolaus-Fiebiger-Center for Molecular Medicine, FAU University Erlangen-Nürnberg, Erlangen, Germany; 11 MRC London Institute of Medical Sciences, Du Cane Road, W12 0NN London, UK; 12 Institute of Clinical Sciences, Faculty of Medicine, Imperial College London, Hammersmith Hospital Campus, Du Cane Road, London, W12 0NN UK

**Keywords:** Embryogenesis, Membrane trafficking, Pluripotent stem cells, Energy metabolism, Pluripotent stem cells

## Abstract

Lima1 is an extensively studied prognostic marker of malignancy and is also considered to be a tumour suppressor, but its role in a developmental context of non-transformed cells is poorly understood. Here, we characterise the expression pattern and examined the function of Lima1 in mouse embryos and pluripotent stem cell lines. We identify that Lima1 expression is controlled by the naïve pluripotency circuit and is required for the suppression of membrane blebbing, as well as for proper mitochondrial energetics in embryonic stem cells. Moreover, forcing Lima1 expression enables primed mouse and human pluripotent stem cells to be incorporated into murine pre-implantation embryos. Thus, Lima1 is a key effector molecule that mediates the pluripotency control of membrane dynamics and cellular metabolism.

## Abstract (web summary)

How pluripotency transcription factors regulate the cellular architecture and energetics has remained largely unknown. Here the authors identify Lima1 as a key effector that mediates the pluripotency control of membrane dynamics and cellular metabolism.

## Introduction

The most frequent types of cancer originate from epithelial cells, which aberrantly activate signalling cascades that promote cell proliferation and allow cells to evade apoptosis^1^. The cancer cells often reprogramme their energy metabolism, lose epithelial morphology and activate invasion and metastasis, enabling a benign adenoma to transform into an invasive carcinoma, which correlates with poor patient prognosis. The malignant transformation is associated with reduced intercellular adhesion as well as cytoskeletal and signalling reorganisation, in a process known as epithelial-to-mesenchymal transition (EMT)^2^.

The process of EMT as well as the activation of several signalling pathways, such as Wnt, Notch and Hedgehog, are common features in both embryonic development and cancer^3^. The EMT, which mediates E-cadherin (E-cad) downregulation, is the driving force enabling malignancy and is also a key step in the process of gastrulation^1^. Similarly, the canonical Wnt/β-catenin (β-cat) signalling is essential for the patterning of the early mouse embryo^4,5^. However, aberrant activation of this pathway can initiate tumorigenesis by promoting the expression of downstream oncogenes^6^.

E-cad and β-cat, together with several actin-binding proteins, such as α-catenin (α-cat) and Lima1 (also known as EPLIN; epithelial protein lost in neoplasm), form the adherens junction (AJ) complex^1,7^. The role of the main components of this complex has been extensively studied in the context of both cancer and embryogenesis, which substantially widens our understanding of their expression pattern, functions and regulation. Yet, it is so far unknown how Lima1, which is considered to be a prognostic marker and tumour suppressor^8^, functions during embryonic development.

From previous studies, the Lima1 locus is known to harbour two promoter regions, which drive the expression of the shorter Lima1-α (600 amino acids) and the longer Lima1-β (759 amino acids) isoforms. Both isoforms contain two actin-binding domains and a central LIM domain^9,10^. Recombinant Lima1-α and -β proteins suppress the depolymerization of actin filaments and cross-link the filaments in bundles^11^. In epithelial cells, the actin fibres are linked to the cadherin-catenin complex (CCC) via α-cat on the AJ^12^. Lima1 can bind α-cat and F-actin, thereby mediating the contact of the CCC with the cytoskeleton^7^ and acting as a mechanosensitive regulator^13^.

In the context of cancer, Lima1 downregulation is implicated in the progression of oral, prostate and breast cancer^10,14–17^. In prostate cancer cells, Lima1 depletion promotes AJ disassembly and cell invasion^18^; conversely, in oesophageal and breast cancer cells, Lima1-α overexpression decreases tumour growth and invasiveness, acting as a tumour suppressor^16,17^. Moreover, Lima1 function is also involved in the cellular metabolism, as shown in the gut, where Lima1 binds to intracellular cholesterol transporters and facilitates cholesterol absorption^19^.

As the majority of studies examining Lima1 have been performed in pathological situations, the role of Lima1 in a developmental context of non-transformed cells is still poorly understood. Therefore, we used the mouse embryo and embryo-derived stem cell lines to analyse the expression pattern and function of Lima1.

Here, we show that Lima1 expression was not confined only to the epithelial tissues of the foetus. During the pre-implantation development, Lima1 was initially enriched in the polar body and later in the pluripotent inner cell mass (ICM) of the early embryo. We determined that Lima1 is under the transcriptional control of the naïve pluripotency circuit and is required for suppressing the formation of membrane blebs in mouse and human pluripotent stem cells. In addition, we found that Lima1 is required for proper mitochondrial function in a cell-autonomous manner and is essential for the growth of solid tumours. Strikingly, ectopic expression of Lima1 in human pluripotent stem cells enabled these cells to be incorporated into murine pre-implantation embryos. Thus, Lima1 is a key effector molecule mediating pluripotency control of membrane dynamics and cellular metabolism.

## Results

### Lima1 expression pattern during mouse embryonic development

**Fig. 1:**
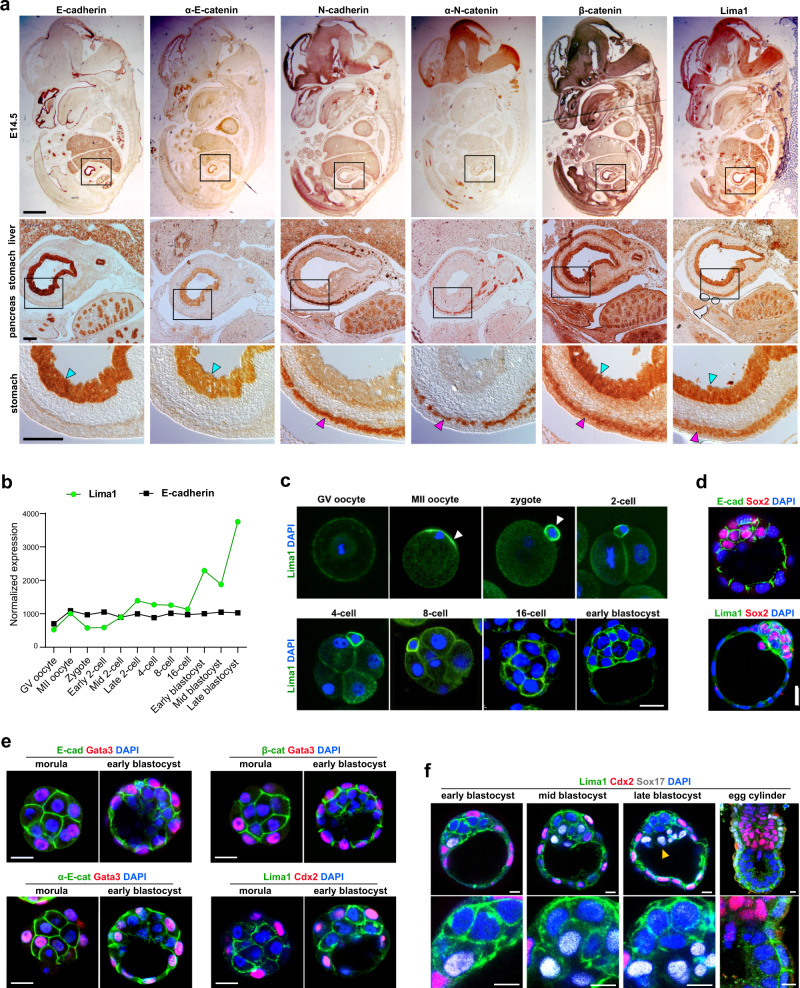
Lima1 expression pattern during mouse embryonic development. **a** E14.5 embryos sectioned and stained for E-cad, α-E-cat, N-cad, α-N-cat, β-cat or Lima1. Blue and magenta arrows indicate epithelial and muscle layers, respectively. **b** Normalized expression levels of Lima1 and E-cad during mouse pre-implantation development, based on Wang et al., 2004. **c** Oocytes and pre-implantation embryos stained for Lima1. Arrowheads indicate Lima1 localisation on the cortical cap in MII oocyte and in the polar body at the zygote stage. **d** Blastocyst stage embryos stained for Sox2 and E-cad or Sox2 and Lima1. **e** Morula and blastocyst stage embryos stained for AJ components, Lima1 and TE lineage markers (Gata3 or Cdx2). **f** Blastocyst and egg cylinder stage embryo stained for Lima1, Cdx2, Sox17 and DAPI. The PE is indicated by an arrowhead. Scale bars, (**a**), 1000 μm, upper panel and 100 μm, lower panels; (**c**), (**d**) and (**e**), 20 μm; (**f**), 10 μm. Experiments were repeated independently at least three times with similar results (**a, c, d, e, f**). Related to Figure S1.

The process of embryogenesis starts from a single cell (zygote), which gives rise to both the embryonic and extraembryonic lineages of the early embryo. After implantation, a complex cascade of cell fate decisions and morphogenetic events form the different tissues and organs of the developing embryo. To analyse Lima1 expression during murine development, we isolated E14.5 embryos, which were sectioned and stained for Lima1. Alongside this, we examined the localisation of the major components of the CCC, namely E-cad, α-E-catenin (α-E-cat), N-cadherin (N-cad), α-N-catenin (α-N-cat) and β-cat. In E14.5 embryos, E-cad and α-E-cat were expressed mainly in epithelial compartments, such as the inner lining of the stomach (Fig. 1a, blue arrowhead). N-cad and α-N-cat were detected in the muscle fibres of the stomach wall (Fig. 1a, magenta arrowhead) as well as in the nervous system, whereas various levels of β-cat were broadly detectable throughout the embryo (Fig. 1a and S1). Although Lima1 is considered to be an epithelial protein^10^, unexpectedly its expression was not confined only to epithelial tissues. Similar to β-cat, Lima1 was broadly expressed, occupying both E-cad and N-cad expression domains (Fig. 1a and S1), suggesting that the potential functions of Lima1 in the developing embryo may span beyond epithelial homoeostasis.

Next, we analysed the expression of Lima1 during the pre-implantation embryogenesis. Using available transcriptomics data^20^, we found that Lima1 mRNA is present in GV and MII oocytes as maternal transcripts, and later the zygotic expression exhibits a sharp increase after the 16-cell stage (Fig. 1b). Staining GV and MII oocytes for Lima1 revealed that the protein is localised in the cytoplasm and the cortex, with strong enrichment in the cortical cup overlying the spindle of the MII oocytes (Fig. 1c). Interestingly, in the zygote, Lima1 accumulated in the extruded polar body, where it remained detectable during the next cleavage stages. Later, Lima1 expression appeared stronger in the inside cells of the 16-cell morula and the ICM of the early blastocyst (Fig. 1c). To validate these observations, we examined the Lima1 expression pattern in combination with lineage markers, discriminating the inner/outer and the ICM/trophectoderm (TE) cells of morula and early blastocyst stage embryos, respectively. In comparison to E-cad, β-cat and α-E-cat, which were overall ubiquitously expressed in both compartments, Lima1 was enriched in the Sox2 +/Cdx2− inner cells of the pre-implantation embryo (Fig. 1d, e).

At mid and late blastocyst stage, we found that the Sox17+ primitive endoderm (PE) cells toned-down Lima1 expression (Fig. 1f, arrowhead), whereas the pre-implantation epiblast (Sox17−/Cdx2−) maintained a relatively high level of Lima1. In addition, Lima1 also became detectable in the TE (Fig. 1f). Conversely, after implantation at early egg cylinder stage (E5.5), Lima1 expression was decreased in the extraembryonic ectoderm (ExE) and the post-implantation epiblast (Fig. 1f). Thus, Lima1 exhibited a dynamic expression pattern in the embryonic and extraembryonic lineages during early mouse development. In the next experiments we focused on examining Lima1 function in the pluripotent lineage.

### Lima1 expression is under the control of the naïve pluripotency circuit

**Fig. 2:**
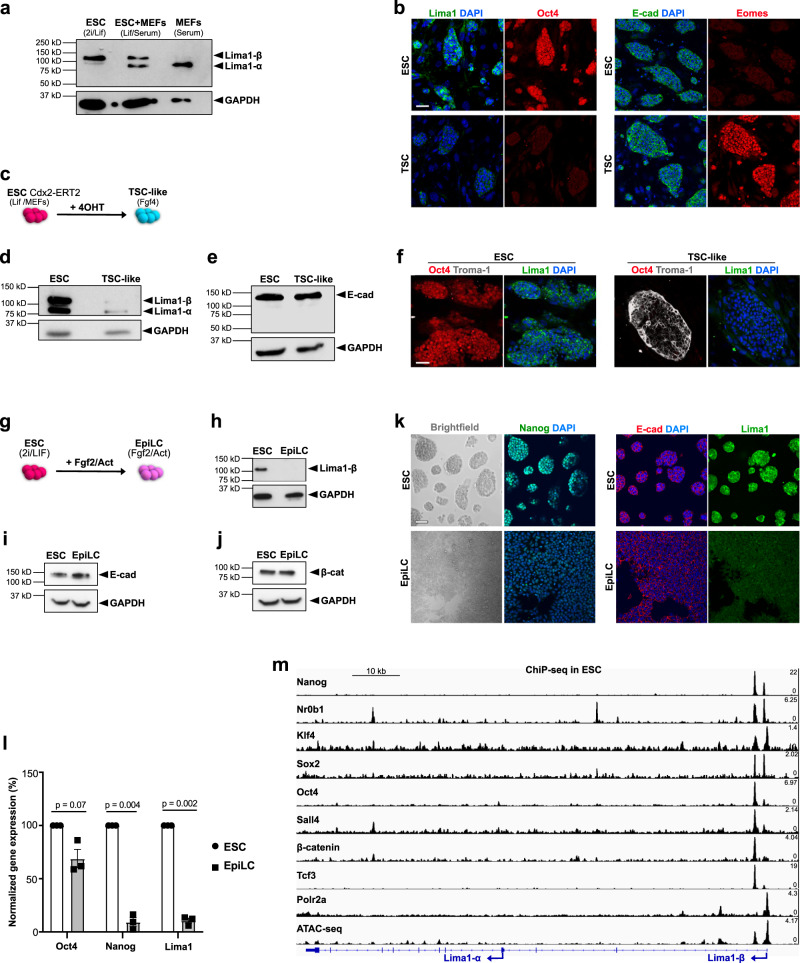
Lima1 expression in embryonic and extraembryonic stem cell lines. **a** Western blot analysis of Lima1 expression in ESC and MEFs. **b** ESC and TSC stained for Lima1 and Oct4 (left panel) or E-cad and Eomes (right panel). **c** Schematic representation of ESC conversion to TSC-like cells. **d** Western blot analysis of Lima1 expression in ESC and TSC-like cells. **e** Western blot analysis of E-cad expression in ESC and TSC-like cells. **f** ESC and TSC-like cells stained for Oct4, Troma1 and Lima1. **g** Schematic representation of ESC conversion to EpiLC. **h** Western blot analysis of Lima1 expression in ESC and EpiLC. **i** Western blot analysis of E-cad expression in ESC and EpiLC. **j** Western blot analysis of β-cat expression in ESC and EpiLC. **k** ESC and EpiLC stained for Nanog (left panel) or E-cad and Lima1 (right panel). **l** Relative expression levels of Oct4, Nanog and Lima1 normalized to GAPDH in ESC and EpiLC. Mean values from three independent repetitions, data represent mean ± SEM, unpaired Student’s *t*-tests, 2-sided. **m** ChIP-seq peaks of pluripotency factors binding on the Lima1 locus in ESC. Polr2a peaks indicate the promoter region, ATAC-seq peaks indicate the accessible chromatin regions. Scale bars, (**b**), (**f**), (**k**), 50 μm. Experiments were repeated independently two times (**a, b, e, f, i, j, k**) or three times (**d, h**) with similar results. Related to Figure S2.

As Lima1 was enriched in the pre-implantation epiblast, we asked whether its expression is maintained in vitro, in embryonic stem cells (ESC). Murine ESC are typically co-cultured with mitotically inactivated mouse embryonic fibroblasts (MEFs) in serum-containing medium, supplemented with leukaemia inhibitory factor (Lif)^21^; alternatively, ESC can be grown without MEFs in serum-free N2B27 medium, supplemented with Lif and 2i (Gsk3 and Mek inhibitors)^22^. In the 2i/Lif culture conditions, we found that the longer Lima1-β isoform was predominantly expressed, whereas, in the presence of MEFs, both Lima1-α and -β isoforms were detectable (Fig. 2a). However, MEFs themselves showed only Lima1-α expression, indicating that Lima1-β is the main isoform present in pluripotent cells (Fig. 2a).

The mouse blastocyst and egg cylinder stage embryos are also a source of trophoblast stem cells (TSC), which are derived from the trophoblast lineage^23^. As Lima1 expression was lower in the TE (early blastocyst) and ExE (egg cylinder), compared to the pre-implantation epiblast, we asked whether TSC and ESC recapitulate this expression pattern in vitro. Similar to the pre-implantation embryo, Lima1 was enriched in ESC but not TSC, whereas E-cad was ubiquitously expressed in both TSC and ESC (Fig. 2b and S2A). In addition, using a forced expression of Cdx2^24^, we reprogrammed the ESC to TSC-like cells (Fig. 2c) and found that, in contrast to the E-cad expression that remained unchanged, Lima1 was downregulated in the TSC-like cells (Fig. 2d–f).

As ESC capture features of the naïve pluripotent lineage of the blastocyst stage embryo^25^, we asked whether Lima1 is under the transcriptional control of the naïve pluripotency network. To test this, we compared Lima1 expression in naïve ESC to more developmentally advanced pluripotent stem cells. Briefly, we treated ESC with Fgf2/Activin, which causes cells to exit naïve pluripotency and transition into so-called epiblast-like cells (EpiLC)^26^ (Fig. 2g). These cells shut down the expression of naïve pluripotency transcription factors, such as Nanog and Esrrb (Fig. 2k and S2B), establishing an early post-implantation epiblast-like state^26^. Similar to Nanog and Esrrb, Lima1 expression was diminished in EpiLC, whereas E-cad and β-cat protein levels remained overall unchanged (Fig. 2h, k and S2B). In addition, Lima1 was also enriched in naïve human induced pluripotent stem cells (naïve hiPSC) in comparison to primed (conventional) hiPSC (Fig. S2C).

During the transition from the naïve to the primed state of pluripotency, Lima1 was substantially downregulated on the mRNA level, exhibiting similar transcriptional dynamics as the naïve pluripotency factor Nanog (Fig. 2l). Moreover, analysis of available ChIP-seq data on pluripotency transcription factor binding in ESC^27–35^ (Table S1) revealed peaks for Nanog, Nr0b1, Klf4, Sox2, Oct4, Sall4, β-cat and Tcf3 in the vicinity of the Lima1-β promoter (Fig. 2m). This suggests that Lima1 is a potential target gene of the naïve pluripotency circuit and may act as a downstream effector, mediating naïve pluripotency control of cytoskeletal dynamics.

### Lima1 controls the membrane dynamics in ESC

**Fig. 3:**
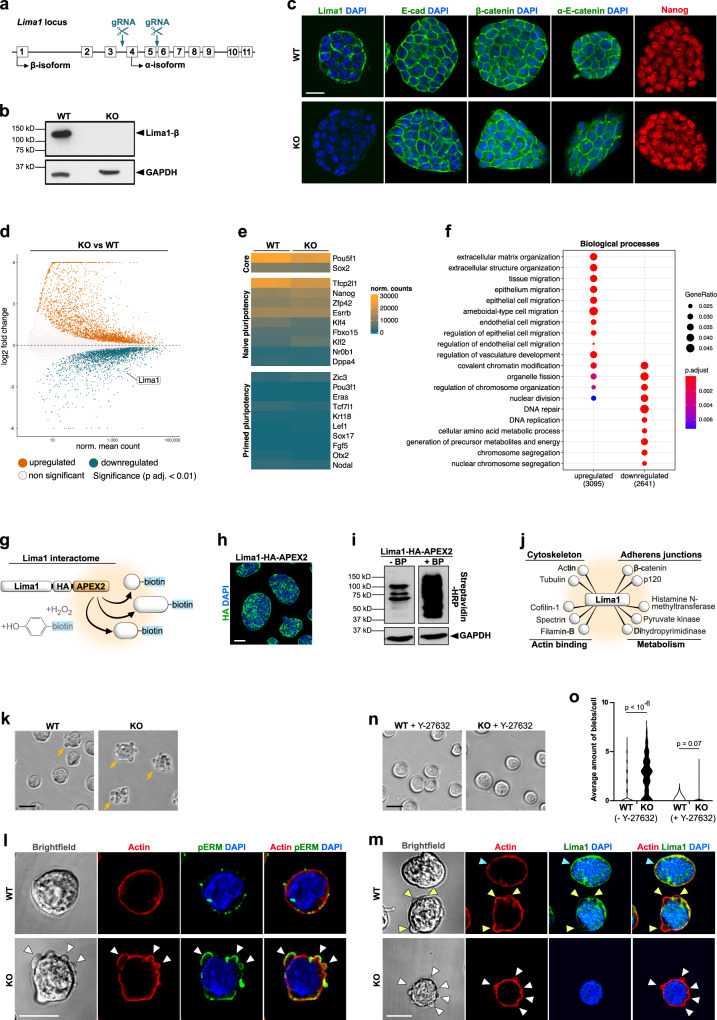
Lima1 depletion in ESC. **a** CRISPR/Cas9 targeting strategy. **b** Validation of Lima1 deletion in ESC by western blot. **c** Lima1 KO and WT ESC stained for Lima1, E-cad, β-cat, α-E-cat or Nanog. **d** MA plot of gene expression in Lima1 KO vs. WT ESC, cultured in N2B27 2i/Lif. Statistical testing for differential gene expression between conditions was performed using the default DESeq2 settings calculating the *p*-value using a two-tailed Wald-test with Benjamini–Hochberg correction for multiple testing and independent filtering of results. **e** Expression of core, naïve and primed pluripotency genes in WT and Lima1 KO ESC. **f** Gene ontology (GO) enrichment analysis “Biological processes”. Gene Ratio indicates the fraction of genes comprised by the GO term, showing significant changes in their expression. **g** Scheme representation of APEX2 interactome analysis. **h** ESC expressing Lima1-HA-APEX2 construct, stained for HA. **i** Western blot analysis of Lima1-HA-APEX2 ESC cultured in the presence of or without biotin-phenol (BP). The biotinylated proteins are detected by streptavidin-HRP. **j** Example of putative Lima1 interaction partners identified by the APEX2 assay. **k** Membrane blebbing (arrows) in WT and Lima1 KO ESC. **l** WT and Lima1 KO ESC stained for actin, pERM and DAPI. Arrowheads indicate membrane blebs. **m** WT and Lima1 KO ESC stained for actin, Lima1 and DAPI. Blue arrowhead indicates Lima1 localisation on the cortex in control ESC; yellow arrowheads indicate loss of Lima1 on the membrane blebs in control ESC; white arrowheads indicate membrane blebs in Lima1 KO ESC. **n** WT and Lima1 KO ESC treated with Y-27632. **o** Mean distribution of cell membrane fluctuation counts per cell over 90 s of time-lapse imaging with 5 s intervals; *n* = 224 (WT), 133 (WT treated), 260 (Lima1 KO), 159 (Lima1 KO treated) cells, Mann–Whitney tests, 2-sided. Scale bar, (**c**) 20 μm; (**h**) 50 μm; (**k**), (**l**), (**n**), (**o**), 10 μm. Experiments were repeated independently two times (**c, h, l, m**) or at least three times (**b, i, k, n, o**) with similar results. Related to Fig. S3.

To analyse Lima1 functions, we established Lima1 knockout (ko) ESC using the CRISPR/Cas9 approach (Fig. 3a). We deleted the genomic region containing exons 4 and 5 to generate a premature stop codon, thus abolishing Lima1 translation (Fig. 3b). Morphologically, Lima1 KO ESC formed typical dome-shaped colonies, undistinguishable from the wild-type (WT) ESC. The main components of the CCC, namely E-cad, β-cat and α-E-cat, were properly localised on the membrane, and Lima1 KO ESC did not exhibit intercellular adhesion defects (Fig. 3c).

To understand whether the loss of Lima1 can affect the status of undifferentiated stem cells, we performed RNA-seq analysis comparing WT and Lima1 KO ESC (Fig. 3d and S3A, Supplementary. Dataset 1). We found no substantial changes in the expression of core, naïve or primed pluripotency markers (Fig. 3e). In addition, Lima1 loss of function did not result in an upregulation of ecto-, endo- or mesodermal markers (Fig. S3B), showing that Lima1 depletion does not promote ESC differentiation.

Interestingly, gene ontology (GO) analysis revealed the enrichment of gene expression associated with epithelial and amoeboid-like cell migration as well as the downregulation of genes involved in cellular metabolism in Lima1 KO ESC. In addition, GO terms such as DNA replication, nuclear division and chromosome segregation were reduced, suggesting that Lima1 KO ESC may exhibit reduced rates of mitosis and potentially a decrease in cell proliferation (Fig. 3f and S3C-S3D). To further explore Lima1 functions, we characterised the Lima1 interactome using APEX2 (peroxidase proximity labelling with ascorbate peroxidase) assay^36^. We fused the Lima1-β isoform (full length) at the C-terminus to an HA-tag, followed by the APEX2 coding sequence (Fig. 3g, h). In the presence of biotin-phenol (BP), a pulse of hydrogen peroxide results in the generation of biotin-phenoxyl radicals by APEX2, which covalently labels proximal endogenous proteins (Fig. 3g, i). The subsequent mass spectrometry analysis revealed four main groups of potential interaction partners: cytoskeletal proteins (e.g., actin and tubulin), actin-binding factors (e.g., cofilin-1 and spectrin), AJ components (e.g., β-cat and p120) and factors involved in metabolism (e.g., pyruvate kinase and dihydropyrimidinase) (Fig. 3j, Supplementary Dataset 2). Among these factors, we found proteins that have been previously shown to directly interact with Lima1, such as actin^37^, or participate together in a larger molecular complex, such as β-cat and p120 catenin^7^. Thus, altogether, the RNA-seq and proteomics analysis suggest that Lima1 might play a role in cytoskeletal dynamics as well as in cellular metabolism.

To examine the potential role of Lima1 in cell migration, we dissociated Lima1 KO and WT ESC and analysed their behaviour on a single-cell level. Strikingly, Lima1 KO ESC exhibited spherical membrane protrusions (blebs), that were rarely detectable in WT cells (Fig. 3k, Movies S1 and S2). The blebbing was exhibited only upon individualisation of the cells and it faded away completely, as the cells came together to form small colonies. Membrane blebbing has been subdivided into three phases, namely initiation (nucleation), expansion and retraction^38^, whereby bleb nucleation is established by cell membrane dissociation from the actin cortex^39^ or via local rupture of the cortex^40,41^. The expansion phase is driven by the hydrostatic pressure of the cytoplasm, followed by the formation of a new actin cortex under the blebbing membrane. Finally, the recruiting of myosin to the newly assembled cortex allows the bleb to retract^38^.

To examine the blebbing in WT and Lima1 KO ESC, we stained the cells for the bleb marker phospho-ERM (ezrin, radixin and moesin)^38^ and found that Lima1 KO ESC exhibit extensive pERM-positive membrane protrusions (Fig. 3L, white arrowheads). In non-blebbing WT ESC, Lima1 was uniformly localised on the cortex, which appeared as a continuous ring (Fig. 3m, blue arrowhead). However, in WT cells that formed blebs, Lima1 was absent from the blebbing membrane (Fig. 3m, yellow arrowheads). Accordingly, genetic deletion of Lima1 converted the overall non-blebbing ESC morphology to a highly blebbing phenotype (Fig. 3k, m and o). This indicates that Lima1 plays an integral role in stabilising the cortex, suppressing the formation of membrane blebs.

In contrast to the naïve ESC, primed pluripotent cells, such as conventional human ESC (hESC) and mouse epiblast stem cells (EpiSC), typically exhibit blebbing upon dissociation into single cells^42^. As bleb formation depends on actomyosin activation via the Rho/ROCK pathway^38^, treatment with a ROCK inhibitor (Y-27632) has been shown to supress the blebbing of primed pluripotent cells^42,43^. Accordingly, Y-27632 treatment also diminished blebbing in Lima1 KO ESC (Fig. 3n, o). Altogether, this shows that Lima1 acts as an effector molecule downstream of the naïve pluripotency circuit, mediating the control of membrane dynamics in ESC.

### Lima1 depletion in ESC results in reduced mitochondrial ATP production, decreased rates of teratoma growth and a reduced contribution to chimeras

**Fig. 4:**
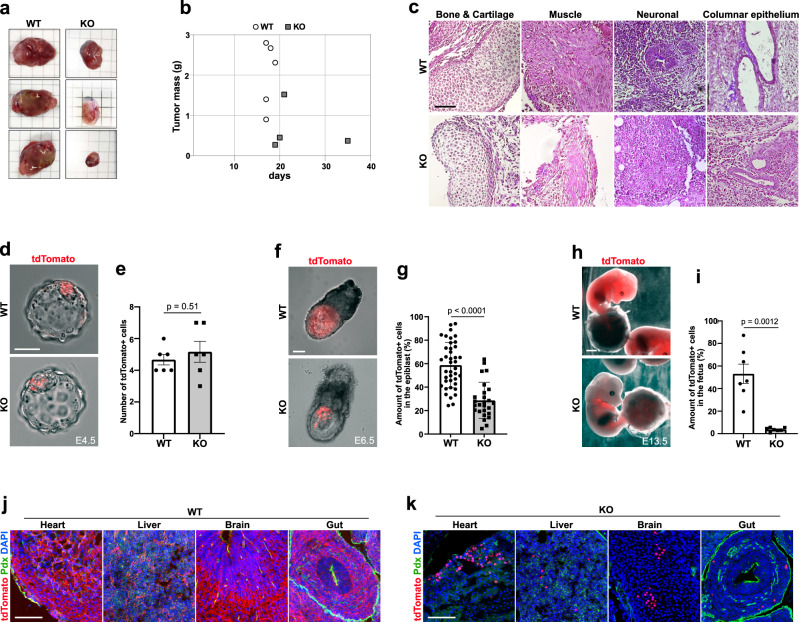
Developmental potential of Lima1 KO ESC. **a** Teratomas derived from Lima1 KO or WT ESC. **b** Growth of WT and Lima1 KO teratomas. **c** Haematoxylin and eosin staining of sectioned teratomas. **d** Chimeric blastocysts containing control (WT) or Lima1 KO donor cells. **e** Quantification of integrated donor cells at blastocyst stage, data represent mean ± SEM, WT *n* = 6 embryos, Lima1 KO *n* = 6 embryos, unpaired Student’s *t*-test, 2-sided. **f** Chimeric E6.5 embryos containing control (WT) or Lima1 KO donor cells. **g** Quantification of the ectopic cells relative to the total epiblast cell number at E6.5. WT (*n* = 40 embryos), Lima1 KO (*n* = 32 embryos), three independent experiments. Data represent mean ± SEM, Mann–Whitney test, 2-sided. **h** Chimeric E13.5 embryos containing control (WT) or Lima1 KO donor cells. **i** Quantification of the ectopic cells relative to the total cell number of the foetus, determined by flow cytometry. WT (*n* = 7 embryos), Lima1 KO (*n* = 7 embryos), three independent repetitions. Data represent mean ± SEM, unpaired Student’s *t*-test, 2-sided. **j** Chimeric E13.5 embryos containing WT donor cells, stained for tdTomato, Podocalyxin and DAPI. **k** Chimeric E13.5 embryos containing Lima1 KO donor cells, stained for tdTomato, Podocalyxin and DAPI. Scale bars, (**c**) 50 μm; (**d**), 20 μm; (**f**) 50 μm; (**h**) 1000 μm; (**j**), (**k**),100 μm; Experiments were repeated independently three times (**c, j, k**) with similar results.

Pluripotent ESC can form non-invasive tumours (teratomas) that consist of derivatives of the three germ layers^44^. Loss of Lima1 has been implemented in malignancy^10,14–17^, but how Lima1 depletion affects the growth of solid tumours is still obscure. To understand whether Lima1 depletion affects the growth rate and/or the composition of solid tumours, we subcutaneously injected WT ESC or Lima1 KO ESC into SCID mice to generate teratomas. Although Lima1 KO cells could give rise to teratomas, their size was substantially smaller compared to the tumours derived from WT ESC (Fig. 4a, b). Nevertheless, Lima1 KO teratomas consisted of tissue derivatives of all three germ layers, indicating that Lima1 depletion does not affect the ESC differentiation capacity (Fig. 4c).

To further explore the behaviour of tissues derived from Lima1 KO ESC, we generated chimeric embryos via aggregation of morulae with nuclear tdTomato-labelled Lima1 KO ESC or membrane tdTomato-labelled WT ESC. At E4.5, we found that similar numbers of Lima1 KO and WT cells were incorporated in the ICM of the chimeric blastocysts (Fig. 4d, e). However, after implantation, at E6.5 and later at E13.5, we found a gradual but substantial decrease of Lima1 KO cells (Fig. 4f–i). Despite the reduced number, the Lima1 KO cells were present in all examined foetal organs (Fig. 4j, k).

**Fig. 5:**
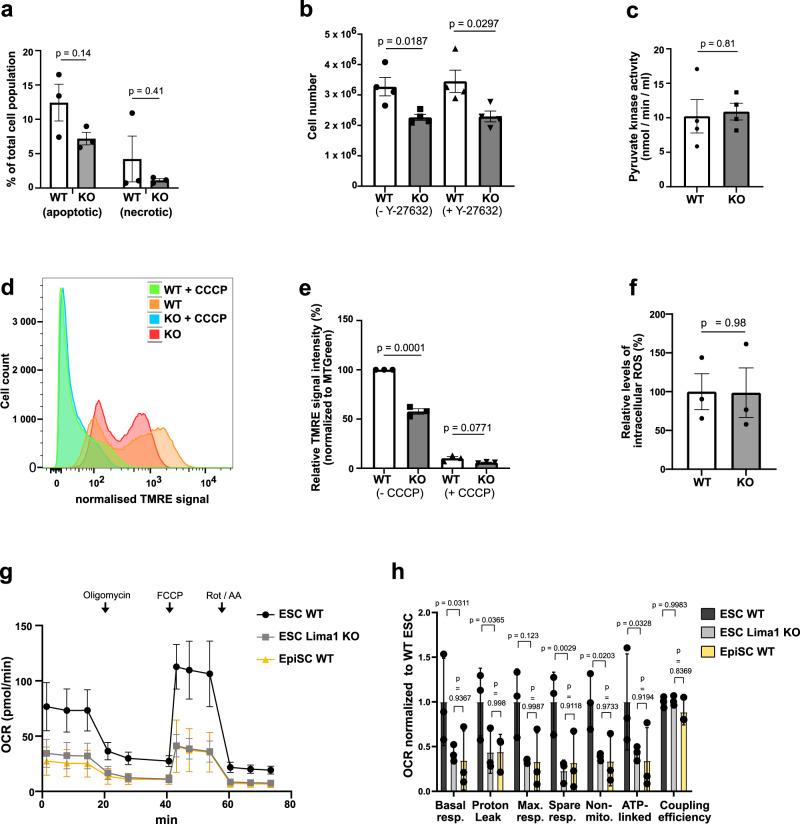
Lima1 is required for proper mitochondrial energetics. **a** Proportion of apoptotic and necrotic Lima1 KO and WT ESC. The cells were individualized by trypsinization and cultured on cell-repellent plates for 2 h at 37 °C. After that, the cell death was determined by annexin V assay in combination with DAPI, three independent experiments, data represent mean ± SEM, unpaired Student’s *t*-test, 2-sided. **b** Quantification of WT and Lima1 KO cell proliferation after 96 h of culture. The cells were treated with or without Y-27632 upon dissociation. Untreated Lima1 KO (*n* = 6 samples) and WT (*n* = 6 samples), three independent experiments. Treated with Y-27632 Lima1 KO (*n* = 6 samples), WT (*n* = 6 samples), four independent experiments. Data represent mean ± SEM, unpaired Student’s *t*-tests, 2-sided. **c** Pyruvate kinase activity measured in WT and Lima1 KO ESC, *n* = 4 independent experiments. Data represent mean ± SEM, unpaired Student’s *t*-test, 2-sided. **d** FACS analysis of WT and Lima1 KO ESC TMRE assay. Plot shows the TMRE signal normalized to the MitoTracker signal. CCCP is a mitochondrial oxidative phosphorylation uncoupler used as a negative control. **e** Quantification of TMRE signal intensity, normalized to MitoTracker signal intensity, *n* = 3 independent experiments. Data represent mean ± SEM, unpaired Student’s *t*-test, 2-sided. **f** Quantification of relative intracellular ROS levels in Lima1 KO ESC, relative to WT ESC. Three independent experiments, data represent mean ± SEM, unpaired Student’s *t*-test, 2-sided. **g** OCR measurement using the Seahorse mitochondrial stress test assay. FCCP−Carbonyl cyanide-4 (trifluoromethoxy) phenylhydrazone; Rot Rotenone, AA−Antimycin. Three independent experiments, mean values ± SEM. **h** Quantification of the OCR analysis, *n* = 3 independent experiments. Data represent mean ± SEM, 2-way ANOVA with Tukey’s multiple comparisons. Related to Fig. S4.

Next, we aimed to determine why the tumour growth and chimerism were reduced in Lima1 KO cells. To understand whether the initial pool of Lima1-deficient pluripotent cells may have survival and/or proliferative disadvantage, we compared the cell death and proliferation of Lima1 KO vs. WT ESC. We found that Lima1 depletion was not associated with an increase in apoptosis or necrosis (Fig. 5a). However, we found a decrease in the proliferation of Lima1 KO ESC cultured either in the presence of or without Y-27632 (Fig. 5b).

As the transcriptional profiling of Lima1 KO ESC and the APEX2 interactome analysis indicated an interplay of Lima1 and cellular metabolism, we asked whether the reduced cell proliferation of Lima1 KO ESC is caused by a metabolic defect. The APEX2 assay identified pyruvate kinase, which catalyses the last step of glycolysis, as a potential interaction partner of Lima1. Nevertheless, we found that pyruvate kinase activity was not altered in Lima1 KO ESC (Fig. 5c).

Previous studies have shown that Lima1 accumulates in RasV12-transformed cells, which results in a decreased mitochondrial transmembrane potential (ΔΨm) in a non-cell-autonomous manner^45–47^. Thus, we asked whether depletion of Lima1 affects the transmembrane potential and energy production of the mitochondria. We analysed the mitochondrial transmembrane potential using the tetramethyl-rhodamine ethyl ester (TMRE) assay and found that the TMRE signal was reduced in Lima1 KO ESC (Fig. 5d, e). As the energy required for ATP production is derived from the ΔΨm^48^, this indicates that Lima1 KO ESC exhibit a decreased energy availability for ATP synthesis in a cell-autonomous fashion. The reduction in the ΔΨm in Lima1 KO ESC did not result in an increase in the intracellular reactive oxygen species (Fig. 5f), suggesting that Lima1 depletion is not associated with mitochondrial oxidative damage.

Next, we conducted a mitochondrial stress test assay using an automatic flux analyser (Seahorse) to examine the oxygen consumption rate (OCR) in WT vs. Lima1 KO cells. This assay measures the OCR before and after applying inhibitors of different electron transport chain (ETC) components, thereby providing an assessment of multiple respiration parameters. We found that the basal and maximal respiration as well as the ATP-linked respiration and the non-mitochondrial oxygen consumption were substantially decreased in Lima1 KO cells (Fig. 5g, h). This shows that Lima1 depletion results in a general reduction of the mitochondrial ATP production rate and energy efficiency in a cell-autonomous manner. As it was previously reported that primed pluripotent cells exhibit low mitochondrial respiration^49^, we also compared the OCR of Lima1 KO ESC to WT EpiSC and found similar levels. Thus, although Lima1 KO ESC maintain the expression of naïve pluripotency transcription factors, they exhibit low mitochondrial respiration akin to the primed pluripotent state.

As the reduction in cell proliferation of Lima1 KO ESC was not compensated by the presence of Y-27632 (Fig. 5b), we examined whether the reduction of the OCR is also Y-27632-independent. Accordingly, we found similar OCR levels in Lima1 KO ESC cultured in the presence of or without Y-27632 (Fig. S4A). In addition, we differentiated WT and Lima KO ESC in vitro, using the embryoid body (EB) assay, and found no substantial difference in the OCR in WT and Lima1 KO EBs (Fig. S4B and S4C). This suggests that the Lima1 loss of function effects on the mitochondrial energetics are context-dependent and are associated with the undifferentiated ESC state.

### Lima1-mediated suppression of membrane blebbing enables the incorporation of primed pluripotent cells into pre-implantation embryos

**Fig. 6:**
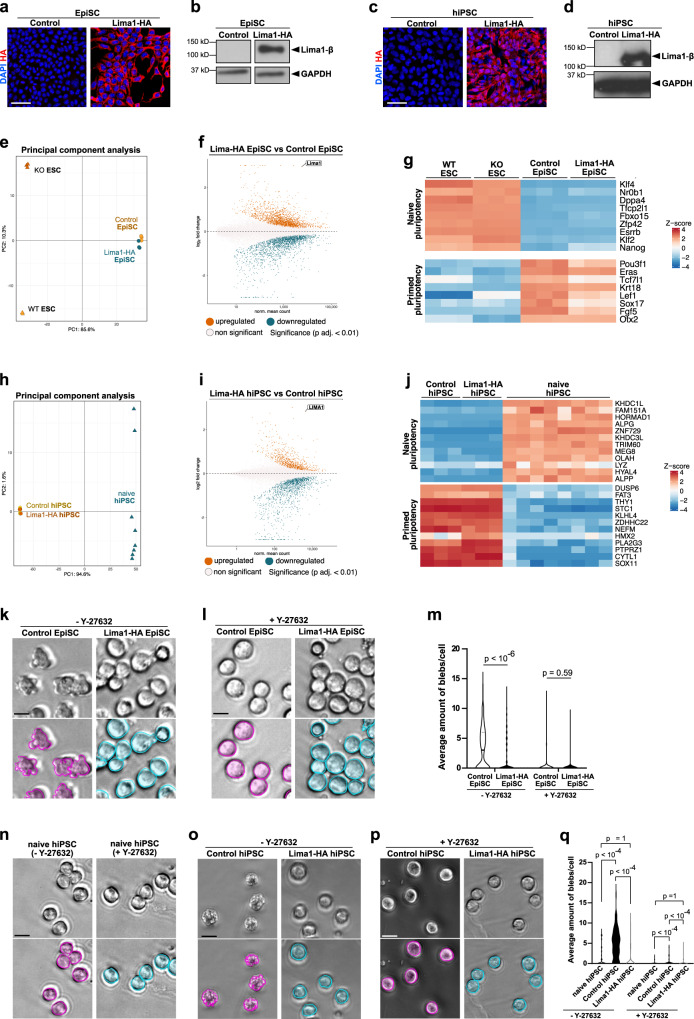
Lima1 ectopic expression in primed pluripotent stem cells. **a** Control and Lima1-HA-expressing EpiLC stained for HA and DAPI. **b** Western blot analysis of Lima1 expression in control and Lima1-HA EpiSC. **c** Control and Lima1-HA-expressing conventional hiPSC stained for HA and DAPI. **d** Western blot analysis of Lima1 expression in control and Lima1 HA conventional hiPSC. **e** Principal component analysis of control EpiSC, Lima1-HA EpiSC, WT ESC and Lima1 KO ESC transcriptomes. **f** MA plot of gene expression in Lima1-HA vs. control EpiSC, two-tailed Wald-test with Benjamini–Hochberg correction for multiple testing and independent filtering of results. **g** Gene expression of naïve and primed pluripotency markers in WT ESC, Lima1 KO ESC, control EpiSC, Lima1-HA EpiSC. **h** Principal component analysis of control conventional hiPSC, Lima1-HA hiPSC and naïve hiPSC (Giulitti et al., 2019)^50^ transcriptomes. **i** MA plot of gene expression in Lima1-HA vs. control conventional hiPSC, two-tailed Wald-test with Benjamini–Hochberg correction for multiple testing and independent filtering of results. **j** Gene expression of naïve and primed pluripotency markers control conventional hiPSC, Lima1-HA hiPSC and naïve hiPSC. **k** Dissociated control and Lima1-HA-expressing EpiSC. **l** Dissociated control and Lima1-HA-expressing EpiSC treated with Y-27632. **m** Mean distribution of cell membrane fluctuation counts per cell over 90 s of time-lapse imaging with 5 s intervals from **k** and **l**; *n* > 177 cells for each condition, Mann–Whitney tests, 2-sided. **n** Dissociated naïve hiPSC cultured without or treated with Y-27632. **o** Dissociated control and Lima1-HA-expressing conventional hiPSC. **p** Dissociated control and Lima1-HA-expressing conventional hiPSC treated with Y-27632. **q** Mean distribution of cell membrane fluctuation counts per cell over 90 s of time-lapse imaging with 5 s intervals from **n, o** and **p**; *n* = 92 (Control hiPSC), 89 (Control hiPSC treated) 88 (naïve hiPSC), 100 (naïve hiPSC treated), 294 (Lima1-HA hiPSC), 245 (Lima1-HA hiPSC treated) cells; 2-way ANOVA with Tukey’s multiple comparisons. Scale bars, (**a**), (**c**), 50 μm; (**k**), (**l**), (**n**), (**o**), (**p**), 10 μm. Experiments were repeated independently two times (**a, b, c, d**) or three times (m, q) with similar results. Related to Fig. S5.

Finally, we examined the gain of function effects of Lima1 expression in mouse and human primed pluripotent stem cells. To this end, we generated murine EpiSC and conventional hiPSC expressing the Lima1-HA construct (β isoform). These cells did not exhibit changes in the level of E-cad expression and adhesion, and they formed colonies that were morphologically undistinguishable from the control lines (Fig. 6a–d, S5A and S5B).

As the endogenous Lima1 is downregulated in primed cells, we asked whether the forced expression of Lima1 in this context results in the activation of naïve pluripotency makers. Therefore, we analysed the transcriptomes of control and Lima1-HA expressing EpiSC using RNA-seq (Fig. 6e–g, S5D–S5F, Supplementary Dataset 3). Principal component analysis revealed that the Lima1-HA EpiSC clustered together with the control EpiSC, apart from the naïve ESC (Fig. 6e). Accordingly, gene expression analysis confirmed the ectopic upregulation of Lima1 (Fig. 6f), which was not associated with an upregulation of naïve pluripotency transcription factors or downregulation of primed pluripotency markers (Fig. 6g).

In addition, we characterised the transcriptional profiles of control (conventional) hiPSC and Lima1-HA expressing hiPSC (Fig. 6h–j, S5G–S5I, Supplementary Dataset 4). We also included in the analysis available transcriptional data of naïve hiPSC^50^. The principal component analysis showed that Lima1-HA and control hiPSC clustered closely together, apart from the naïve hiPSC (Fig. 6h). Similar to the Lima1-HA EpiSC, the ectopic expression of Lima1 in conventional hiPSC was not associated with activation of naïve pluripotency genes or downregulation of primed pluripotency factors (Fig. 6i, j). Altogether, this shows that the expression of Lima1-HA in both mouse and human primed pluripotent stem cells does not promote conversion to naïve pluripotency.

Next, we asked whether the ectopic expression of Lima1 in primed cells affects membrane blebbing. Control EpiSC and hiPSC formed blebs (Fig. 6k, o), which were efficiently suppressed by Y-27632 treatment (Fig. 6l, p), in agreement with previous reports^42,43^. In contrast, blebbing was rarely observed in naïve hiPSC (Fig. 6n). We found that formation of blebs was substantially reduced as a result of the ectopic Lima1 expression in EpiSC and conventional hiPSC (Fig. 6k–m, o–q). In addition, studies have reported that the membrane blebbing in conventional human ESC (hESC) triggers an apoptotic response, whereas treatment with a ROCK inhibitor protects the dissociated hESC from cell death^42,43^. Accordingly, we found that suppressing membrane blebbing by Lima1 enhanced the survival of conventional hiPSC (Fig. S5C).

**Fig. 7:**
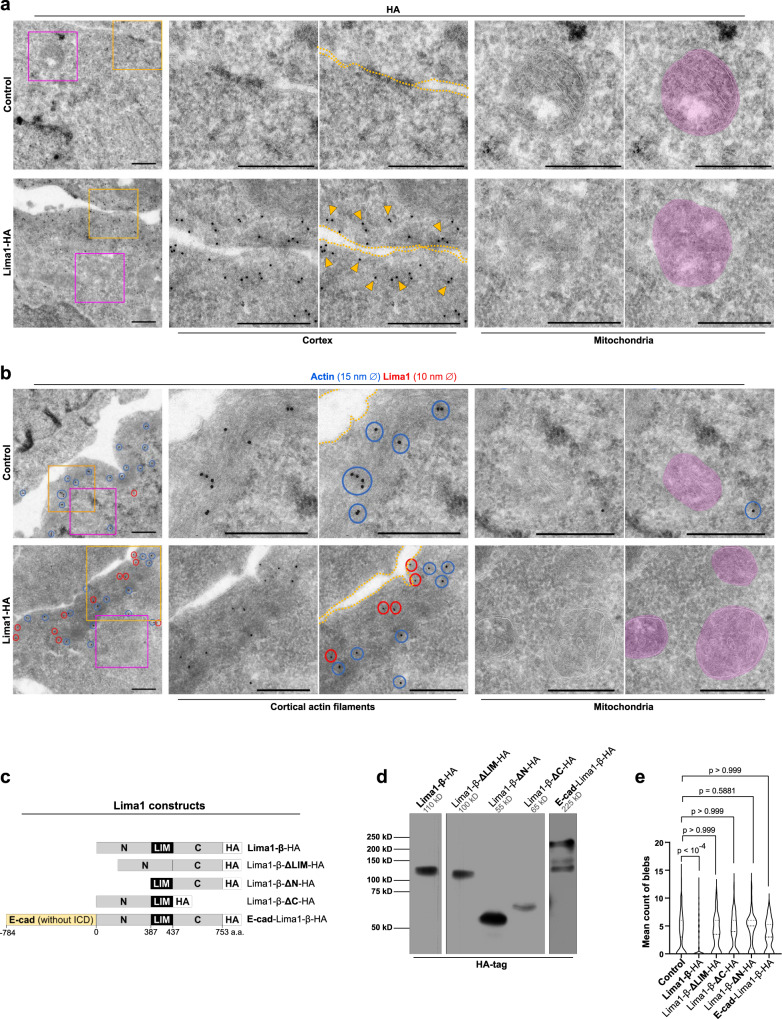
Ultrastructural analysis and expression of Lima1 deletion constructs in EpiSC. **a** Transmission electron microscopy of immunogold staining for the HA-tag in control and Lima-HA EpiSC. Arrowheads indicate the HA signal; mitochondria are marked in magenta. At least three technical replicates with similar results. **b** Transmission electron microscopy of double immunogold staining for Lima1 and Actin in control and Lima-HA EpiSC. Blue and red circles indicate the 15 nm and 10 nm gold particles, respectively; mitochondria are marked in magenta. At least three technical replicates with similar results. **c** Maps of the Lima1 constructs. **d** Western blot analysis of the expression of the Lima1 constructs detected by anti-HA antibody. **e** Mean distribution of cell membrane fluctuation counts per cell over 90 s of time-lapse imaging with 5 s intervals; *n* = 225 (Control), 245 (full-length Lima1-β), 68 (del LIM domain), 78 (del C-terminus), 51 (del N-terminus), 18 (E-cad-Lima1-β fusion) cells, one-way ANOVA. Scale bars, (**a**), (**b**) 1 μm. Related to Fig. S6.

To further examine the effects of Lima1 expression on the membrane dynamics, we performed ultrastructural analysis to determine the subcellular localisation of Lima1 using immunogold labelling. We stained control and Lima1-HA EpiSC for the HA-tag and found Lima1-HA signal on the cell cortex (Fig. 7a). We did not detect enrichment of Lima1 in the mitochondria (Fig. 7a, b), suggesting that Lima1 is not directly involved in the mitochondrial composition. In addition, we examined the OCR in WT EpiSC or WT hiPSC vs. Lima-HA EpiSC or Lima-HA hiPSC, respectively and found no substantial difference between the control and Lima1-HA expressing cells (Fig. S6A–S6D), indicating that the effects of Lima1 on the mitochondrial energetics are associated with the naive pluripotent state.

To understand whether Lima1 is localised within the cortical actin, we carried out double immunogold staining using two different sizes of gold particles— 10 nm of diameter for detecting Lima1 and 15 nm of diameter for detecting actin. The transmission electron microscopy confirmed that Lima1 was enriched on the cortical actin filaments (Fig. 7b), in accord with the proposed role of Lima1 in stabilisation of the cortex, supressing the formation of membrane blebs.

Lima1 protein harbours two actin-binding domains localised in the N-terminal and C-terminal regions, as well as a central LIM domain, which was suggested to mediate Lima1 dimerization^8–10^. To examine the role of these domains on the membrane dynamics, we generated deletion constructs, lacking the N-terminal or the C-terminal regions, or the LIM domain and expressed these constructs in EpiSC (Fig. 7c, d). In comparison to the full-length Lima1-HA, N- and C-terminal deletions, as well as monomeric Lima1 (ΔLIM), failed to suppress membrane blebbing (Fig. 7e). Likewise, tethering Lima1 to the cell membrane by fusing the full-length Lima1 to E-cad (lacking the intracellular domain) was also inefficient to abolish blebbing (Fig. 7c–e). This indicates that the cytoplasmic pool of dimeric Lima1 proteins, bound to the cortical actin filaments, suppresses the formation of membrane blebs in pluripotent stem cells.

**Fig. 8:**
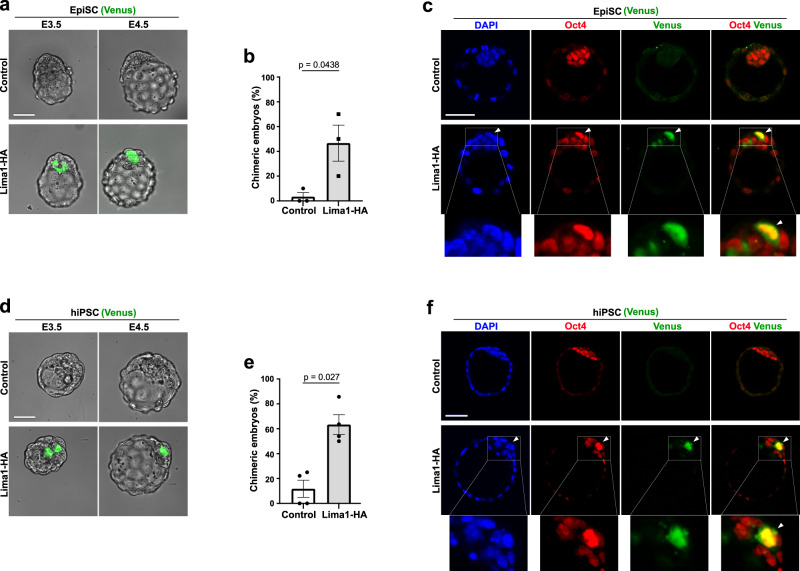
Engraftment of Lima1-HA-expressing primed pluripotent cell in mouse pre-implantation embryos. **a** E3.5 and E4.5 chimeric blastocysts generated via morula aggregation using control or Lima1-HA-expressing EpiSC. **b** Quantification of the chimeric blastocysts containing control (22 embryos) or Lima1-HA-expressing (26 embryos) EpiSC at E4.5. Data represent mean ± SEM, *n* = 3 independent experiments, unpaired Student’s *t*-test, 2-sided. **c** Chimeric blastocysts generated via morula aggregation using control (upper panel) or Lima1-HA-expressing EpiSC (lower panel) and stained for Venus, Oct4 and DAPI. Arrowhead indicates integrated EpiSC. **d** E3.5 and E4.5 chimeric blastocysts generated via morula aggregation using control or Lima1-HA-expressing conventional hiPSC. **e** Quantification of the chimeric blastocysts containing control (30 embryos) or Lima1-HA-expressing (33 embryos) conventional hiPSC at E4.5. Data represent mean ± SEM, *n* = 4 independent experiments, Mann–Whitney test, 2-sided. **f** Chimeric blastocysts generated via morula aggregation using control (upper panel) or Lima1-HA-expressing conventional hiPSC (lower panel) and stained for Venus, Oct4 and DAPI. Arrowhead indicates integrated hiPSC. Scale bars, (**c**), (**f**), 20 μm. Experiments were repeated independently three times (**a, c, d**) with similar results. Related to Fig. S7.

The establishment of a primed pluripotent state also results in a reduced incorporation efficiency of EpiSC and hiPSC into pre-implantation embryos. To understand whether suppressing the membrane blebs via forced expression of Lima1 allowed for EpiSC integration into early embryos, we aggregated control or Lima1-HA EpiSC with 8-cell stage morulae. We found a substantial increase in the number of chimeric blastocysts when we used donor EpiSC expressing Lima1-HA for aggregation (Fig. 8a–c). Moreover, ectopic upregulation of Lima1 in conventional hiPSC enabled the generation of chimeric blastocysts (Fig. 8d–f). Alternatively, supplementing the embryo culture medium used for morula aggregation with a ROCK inhibitor allowed the control EpiSC or hiPSC to be incorporated into pre-implantation embryos (Fig. S7A–S7D). Altogether, this shows that suppression of membrane blebbing by Lima1 is sufficient to enable primed pluripotent cells engraftment into murine pre-implantation embryos.

## Discussion

Lima1 is an epithelial actin-binding protein that has been implicated and extensively studied in the progression of various types of cancers^8^. It also binds α-cat and associates with the AJ complex on the cell membrane^7,13^. We found that in the context of the developing mouse embryo, Lima1 exhibits a pattern of expression that is not confined only to epithelial compartments.

A typical feature of the AJ complex proteins E-cad and N-cad is their almost mutually exclusive expression pattern. Similarly, α-E-cat and α-N-cat are preferentially found in complex with E-cad and N-cad, respectively, whereas β-cat exhibits very broad expression, as it binds directly to both cadherins and also plays a central role in the Wnt signalling pathway. During EMT, E-cad is downregulated and N-cad upregulated in a process known as cadherin switch^1^. Unexpectedly, we found Lima1 localisation in both E-cad and N-cad expression domains, overall resembling the β-cat expression pattern. However, our immunohistochemistry analysis does not differentiate between the long or short isoforms of Lima1. Therefore, Lima1-α and Lima1-β may still exhibit an unaccounted differential expression pattern in the foetus. Moreover, we found β-cat and Tcf3 binding on the Lima1-β promoter region, which indicates that in certain contexts, Lima1-β could be a direct target of the Wnt pathway. As Wnt signalling is aberrantly activated in cancer, this may help explain why upregulation of Lima1-β is observed in some breast cancer cells lines, where the shorter isoform is depleted^10^.

We found that the Lima1 transcripts and protein are already present in the oocyte before fertilisation. During the transition from the GV to MII stage, Lima1 accumulates on the membrane above the spindle and later in the polar body. This specialised membrane domain, known as cortical actin cup, plays a critical role in the asymmetric division during meiosis, which results in the extrusion of the polar body^51^. Several actin nucleators, such as ARP2/3 complex and formin-2, regulate the cytoskeletal remodelling during oocyte maturation^52,53^, and it would be interesting to address the potential role of Lima1 in this process, which would require depleting the maternal transcripts. Interestingly, Lima1 has already been shown to localise in the cleavage furrow in HeLa cells, where it is involved in the maintenance of the contractile ring^54^. Therefore, Lima1 might also play a role during meiosis, particularly in the process of the polar body extrusion.

**Fig. 9:**
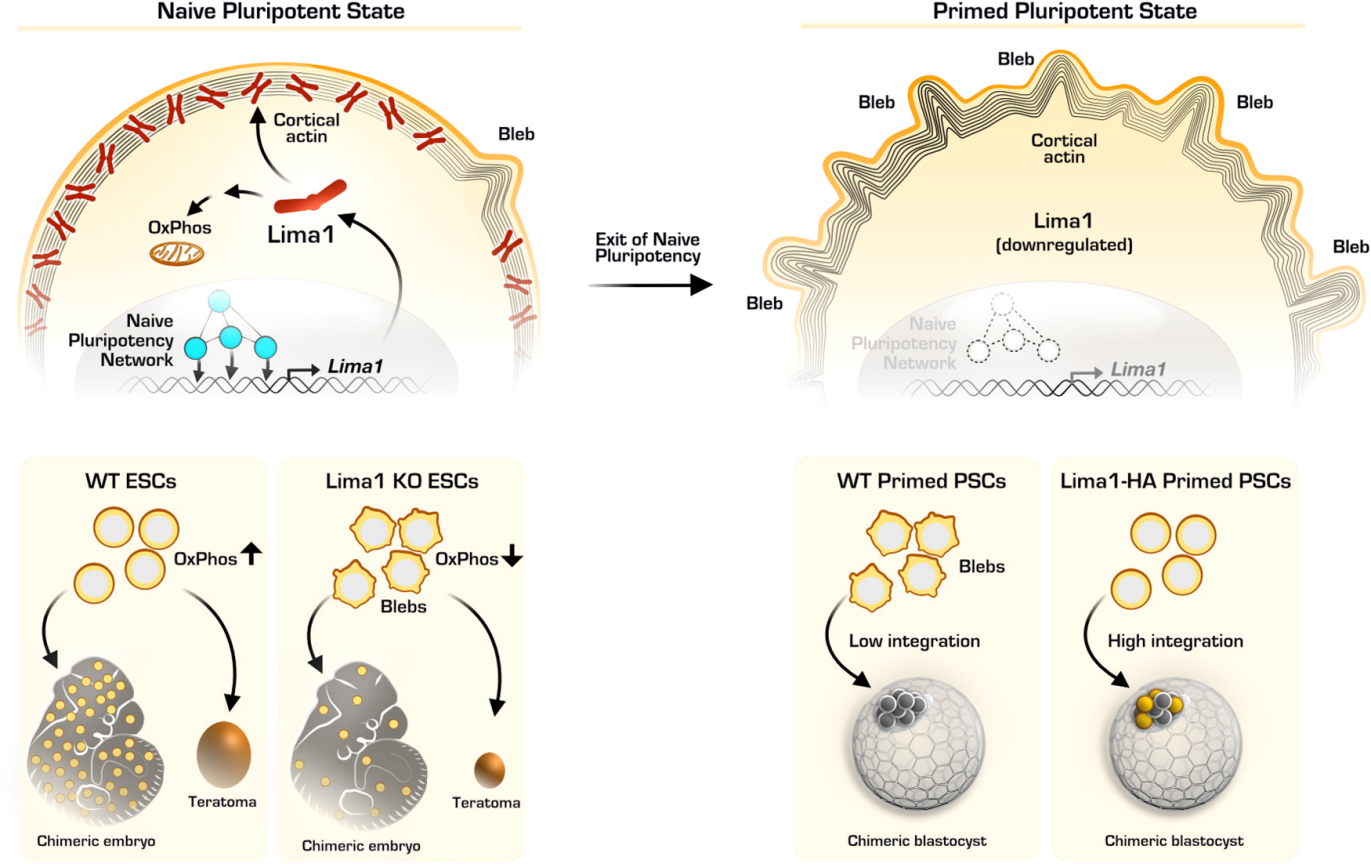
Lima1 mediates the pluripotency control of membrane dynamics and cellular metabolism. Lima1 expression is promoted by the naïve pluripotency transcription factors, which occupy the Lima1 promoter region. In turn, the cytoplasmic pool of dimeric Lima1 proteins bound to the actin filaments stabilises the cortex, thereby suppressing the formation of membrane blebs in naïve pluripotent cells. Lima1 is also involved in the mitochondrial energetics and is crucial for growth of teratomas and embryonic chimerism of ESC. Upon exit of naïve pluripotency, the naïve transcriptional circuit is dismantled and Lima1 is downregulated, resulting in the formation of membrane blebs. Accordingly, ectopic expression of Lima1 in mouse and human primed pluripotent cells suppresses membrane blebbing and enables the incorporation of the primed cells into murine pre-implantation embryos. OxPhos, oxidative phosphorylation; naïve pluripotency transcription factors are marked in blue; donor cells are marked in yellow.

At the 16-cell stage, we found that Lima1 expression is enriched in the inside cells of the morula. These cells give rise to the ICM of the blastocyst, whereas the outside cells form the TE^55^. In contrast, E-cad, β-cat and α-E-cat exhibited overall uniform expression in the early lineages. Accordingly, Lima1 was also highly expressed in ESC, whereas E-cad and β-cat showed similar levels of expression in both ESC and TSC. Moreover, E-cad and β-cat expressions were not affected by the exit of naïve pluripotency and the establishment of a primed state, whereas Lima1 was downregulated in response of this transition. Essentially, we found that the Lima1 promoter region is bound by several pluripotency transcription factors, such as Nanog, Nr0b1, Klf4, Sox2, Oct4, Sall4, as well as β-cat and Tcf3, indicating that Lima1 expression is under the control of the naïve pluripotency network (Fig. 9). This suggests that the establishment of the pluripotent fate activates Lima1 expression in the inner cells of the early embryo.

At mid and late blastocysts stage, we found that the epiblast cells retain a relatively high level of Lima1, whereas the PE cells appeared to decrease Lima1 expression. A recent study of Yanagida et al. showed that the PE cells exhibit larger surface fluctuations (membrane blebs), compared to the epiblast cells. Based on these results, a model was proposed suggesting that the difference in the membrane dynamics is involved in the segregation of PE and epiblast cells during blastocyst maturation^56^. The PE cells displayed also elevated levels of pERM^56^, similarly to our observations of pERM enrichment in the ameboid membrane protrusions in Lima1 KO ESC. Thus, a reduction of Lima1 expression may serve as an indicator of cells that exhibit dynamic membrane fluctuations. Moreover, the progressive changes in the expression pattern of Lima1 in the PE and epiblast cells of the ICM may contribute to a surface fluctuations-driven segregation of these lineages, which as proposed by Yanagida et al., resembles phase separation between active and passive particles in colloidal mixtures^56^.

Loss of Lima1 did not affect the expression of pluripotency markers nor the differentiation capacity of the ESC. However, the growth of the Lima1 KO teratomas, as well as the proportion of donor Lima1 KO cells in post-implantation embryos, were severely reduced. ESC exhibit bivalent metabolism, using both OxPhos and glycolysis, whereas somatic cells rely mainly on OxPhos for ATP production to support their growth^57^. In addition, the transition from a naïve to a primed state of pluripotency results in the reduction of OxPhos and a switch mainly to glycolysis^49,58,59^. Loss of Lima1 did not cause cells to enter into primed pluripotency, but the mitochondrial ATP production rate was substantially reduced. Accordingly, Lima1 KO ESC exhibited decreased proliferation rate, which may contribute to the overall smaller size of the teratomas. The exact underlying molecular mechanism remains open. As Lima1 exhibited a broad expression in the foetus, loss of Lima1 may affect actin dynamics, membrane properties and adherens junctions in various tissues, which may also account for the reduced tumour growth and chimerism. In addition, Lima1 KO cells might face cell competition and potentially be outcompeted by the host embryo’s cells, as a recent study showed that cells with mitochondrial dysfunction are eliminated in the post-implantation conceptus^60^.

Previously, Zhang et al. reported a conditional deletion of exon 5 of the Lima1 locus via adenoviral EIIa promoter-directed Cre recombinase^61^ for the generation of heterozygous mice, which exhibited lower cholesterol absorption and decreased plasma total cholesterol levels^19^. The homozygous exon 5 del/del mice were viable and also displayed decrease cholesterol absorption^19^. Here, in addition to exon 5, we deleted also exon 4, which contains the second transcription start site of the Lima1 locus. Whether this targeting strategy may result in an embryonic phenotype remains to be determined. Alternatively, Lima1 KO cells may exhibit a developmental disadvantage in vivo only in a competitive environment, such as in the context of chimeric embryos.

Understanding how Lima1 regulates mitochondrial function on a molecular level will require further investigation. Although we found several putative interaction partners involved in cellular metabolisms, such as pyruvate kinase and dihydropyrimidinase, none of these candidates directly control any key steps of energy homeostasis. Thus, it is possible that a yet unidentified Lima1 interaction partner(s) is involved in this process. Alternatively, the primary function of Lima1 as an actin-binding protein may indirectly affect the cellular metabolism. For instance, it has been shown that actin cytoskeleton dynamics regulate the activity of aldolase A, which is a key glycolytic enzyme^62^. In addition, application of force to the E-cad complex promotes the activity of AMP-activated protein kinase (AMPK), which plays a critical role in glucose and fatty acid uptake and oxidation^63^. As Lima1 can act as a mechanosensor^13^, potential changes in the mechanotransduction in Lima1 KO cells may also, in turn, influence the cellular metabolism.

Lima1 KO ESC exhibited membrane blebbing similarly to primed pluripotent cells, where the endogenous Lima1 expression was downregulated upon exiting the naïve state (Fig. 9). Ectopic expression of Lima1 in EpiSC and conventional hiPSC was sufficient to stabilise the actin cortex and prevent blebbing nucleation. A stabilising role of Lima1 on the cortex has also been reported in human umbilical vein endothelial cells (HUVEC), where downregulation of Lima1 results in gaps on the cortical actin^64^. Interestingly, HUVEC form classical lamellipodia and junction-associated intermittent lamellipodia in which Lima1-α controls the protrusion dynamics, whereas Lima1-β binds and stabilizes stress fibres^37^. Moreover, it was reported that Erk-mediated phosphorylation of Lima1 reduces the affinity for actin filaments, which contributes to cell motility^65^. In 2i/Lif culture conditions, Erk activity is suppressed by the chemical inhibition of the upstream Mek, whereas in primed pluripotency culture conditions, Erk activity is promoted by the stimulation of the Fgf signalling^22,26^. Thus, although, Lima1 is transcriptionally downregulated upon exiting naïve pluripotency, we cannot exclude that additional fine-tunning of Lima1-actin interactions occur on a post-translational level by the Fgf/Erk pathway.

We found that the Lima1-mediated suppression of membrane blebbing enabled primed pluripotent cells to be integrated into murine pre-implantation embryos. Interestingly, E-cad overexpression has also been shown to promote EpiSC incorporation^66^, but we found no substantial changes in the endogenous E-cad level upon exit of naïve pluripotency or upon ectopic expression of Lima1 in EpiSC. Nevertheless, the stabilisation of the actin cortex by Lima1 gain of function may promote more stable F-actin engagement with the E-cad complex, thus resembling the net effect of E-cad overexpression. In addition, it has been shown that human pluripotent stem cells can be integrated into pre-implantation mouse embryos via suppression of cell death^67–69^. Accordingly, Lima1 expression, as well as treatment with a ROCK inhibitor, promoted the survival of dissociated hiPSC. This indicates that downregulation of the endogenous Lima1 in primed hiPSC, which results in membrane blebbing and apoptotic response, is a key part of the interspecies engraftment barrier.

In tumour cells, membrane blebbing can mediate cell motility, which is used by the malignant cells as an alternative mechanism of migration^41,70^. This alteration in the morphology and behaviour of the cancer cells is known as a mesenchymal–amoeboid transition (MAT)^70^. As blebbing motility does not require degradation of the extracellular matrix, MAT allows tumour cells to evade anti-cancer treatments, which rely on pharmacological protease inhibitors^70,71^. Our results in pluripotent cells show that Lima1 depletion enables blebbing and suggest that in pathological conditions, this may promote tumour resistance to protease inhibitors. Thus, treating malignancies associated with Lima1 deficiency may benefit from cancer therapeutics that target regulators of membrane blebbing.

## Methods

### Mice

Animal experiments and husbandry were performed according to the German Animal Welfare guidelines and approved by the Landesamt für Natur, Umwelt und Verbraucherschutz Nordrhein-Westfalen (State Agency for Nature, Environment and Consumer Protection of North Rhine-Westphalia), protocol number 84-02.04.2016.A186. The mice used in the study were at ages ranging from 6 weeks to 5 months. The animals were maintained at 21.5 °C, 55–60% humidity under a 14-h light/10-h dark cycle with free access to food and water. Male mice were kept individually, whereas the female mice were housed in groups of up to four per cage. Embryos for experiments were obtained from wild-type CD1 and B6C3F1 from matings using females with natural ovulation cycles or after superovulation.

### Cell culture

DR-4 or CF-1 MEFs (gift from Prof. Dr. Hans R. Schöler) were cultured on gelatin-coated plates in DMEM medium (Sigma, D5671), supplemented with 15% FCS (Biochrome, S0615), 10 U/ml penicillin/50 μg/ml streptomycin solution (Sigma, P4333), 2 mM L-glutamine (Sigma, G7513), 1 mM sodium pyruvate (Sigma, S8636), 1x MEM non-essential amino acids (Sigma, M7145) and 0.15 mM β-mercaptoethanol (Sigma, M3148). MEFs were mitotically inactivated by exposure to 10 μg/ml mitomycin C (Sigma, M0503) for 2 h, before splitting for co-culture experiments.

Mouse ESC—E14 (gift from Prof. Dr. Hans R. Schöler), R1 (gift from Prof. Dr. Rolf Kemler) or mT/mG^72^, were grown on DR-4 feeders in MEF medium supplemented with 1000 U/ml Lif (home-made). The cells were split every 2–3 days using 0.25% Trypsin-EDTA (Gibco, 25200056). For experiments requiring ground state culture conditions, the ESC were cultured on human plasma fibronectin coated plates (Millipore; FC010) in N2B27 medium consisting of 48% DMEM F-12 medium (Invitrogen, 21331-046), 48% Neurobasal medium (Invitrogen, 21103-049), 1% B27 Supplement w/o vitamin A (Invitrogen, 12587-010), 10 U/ml penicillin/50 μg/ml streptomycin solution (Sigma, P4333), 2 mM L-glutamine (Sigma, G7513), and 0.15 mM β-mercaptoethanol (Sigma, M3148), supplemented with 1000 U/ml Lif (home-made), 3 μM CHIR99021 (Biomol, 13122) and 0.4 μM PD0325901 (Biomol,13014).

ESC grown in ground state culture conditions were converted to EpiLC^26^ by 48 h culture in N2B27 medium supplemented with 1% KSR (Knockout Serum Replacement, Gibco, 10828-028), 20 ng/ml activin A and 12 ng/ml Fgf2.

TSC^73^ were maintained on MEFs in MEF medium supplemented with 25 ng/ml Fgf4 (Peprotech, 100-31) and 1 μg/ml Heparin (Sigma, H3393). ESC were converted to TSC-like cells via forced expression of Cdx2-ERT2 construct^24^, following treatment with 0.5 μM 4-Hydroxytamoxifen (Sigma, H7904-5MG).

E3 EpiSC^74^ were cultured in medium containing 50% N2B27 and 50% MEF-conditioned medium, consisting of DMEM F-12 supplemented with 20% KSR, penicillin-streptomycin, 2 mM L-glutamine, 1x non-essential amino acids and 0.15 mM β-mercaptoethanol. The EpiSC medium was supplemented with 10 ng/ml activin A and 5 ng/ml Fgf2 before use. The cells were split every 2–3 days using accutase (Sigma, A6964).

Conventional hiPSC^75^ were cultured on matrigel coated plates in MEF-conditioned medium, supplemented with 10 ng/ml Fgf2. The cells were split every 3 days using TrypLE Express (Gibco, 12604-013).

Naïve hiPSC^76^ were cultured in PGXL medium consisting of 48% DMEM F-12 medium, Neurobasal (48%) medim, 1% B27 Supplement (Invitrogen, 17504044), 10 U/ml penicillin/50 μg/ml streptomycin solution, 2 mM L-glutamine and 0.15 mM β-mercaptoethanol, supplemented with 1 μM PD0325901, 2 μM Gö6983 (Tebu-bio, 800088), 2 μM XAV939 (Sigma, X3004) and 250 U/ml Lif. The cells were split every 3–4 days using Accutase and cultured on geltrex coated plates (Thermo Fischer Scientific, A1413302).

Xfect (Takara, 631320) or Lipofectamine 2000 (Invitrogen, 11668027) were used for transfection, following the manufacturer’s instructions.

### Generation of Lima1 KO ESC via CRIPSR/Cas9-mediated deletion

Guide RNAs (guide RNA 1 5'-3': CTGCTCACTTTGTCCTTATA, guide RNA 2 5'-3': GTCGCATGTTAGCTGCAAGA) were designed using the MIT tool (www.crispr.mit.edu). Each of the guide RNA coding sequences was synthesised as a pair of complementary oligonucleotides, which were annealed and separately cloned into pSpCas9(BB)-2A-Puro (PX459) vectors^77^. The vectors were additionally modified to express fluorescent reporters (GFP or Cerulean) in order to facilitate the sorting and collection of double-positive cells. After re-seeding, individual ESC clones were manually picked and expanded. Homozygous deletion of Lima1 was confirmed by PCR genotyping (primer pair 1 5'-3': TCTTGTTGTTTGTGGCATAC and CACTCACTTTCCTAACATTGA—653 bp product of Lima1 KO allele; primer pair 2 5'-3': GTTTTATCACTTCCTGCTTCA and AAAAGACATACTGTCCACACA —796 bp product of WT Lima1 locus), as well as by sequencing and western blot analysis.

### Fluorescence-activated cell sorting (FACS)

Adherent cells were dissociated and resuspended in 3% FCS/PBS solution (FACS buffer). Chimeric E13.5 embryos were mechanically homogenized, and the cells were subsequently dissociated using trypsin. The cell suspension was pelleted by centrifugation and resuspended in FACS buffer. Single viable cells were selected based on FSC- and SSC-gating. The FACS gating plots are presented in Fig. S8. Cell sorting and analysis was performed using FACSAria IIIu and FACSAria Fusion systems, equipped with FACSDiva Software (v8.01). FlowJo software (v10.7.1) was used for data analysis and plotting.

### Immunofluorescence labelling and confocal microscopy

Adherent cells were grown on 8-well μ-slide plates (Ibidi, 80826). Non-adherent cells were collected and pelleted by centrifugation. After washing with PBS, the cells were fixed using 4% PFA for 15 min at room temperature and then permeabilized using 0.3% Triton X-100/PBS for 5 min. After washing, the samples were incubated with blocking buffer (3% BSA/PBS) for at least 30 min, and after that, the primary antibodies diluted in blocking buffer were applied to the sample and incubated overnight at 4 °C. On the next day, the primary antibody solution was removed, the cells were washed and incubated with secondary antibodies diluted in blocking buffer. Nuclei were counterstained with DAPI.

Pre-implantation embryos were fixed using 4% PFA/PBS for 10 min, whereas egg cylinder stage embryos were fixed for 15 min. The samples were permeabilised using 0.3% Triton X-100/PBS solution supplemented with 100 mM glycine for 5 min (pre-implantation embryos) or 15 min (post-implantation embryos). After washing with 5% FCS/PBS, the samples were incubated with primary antibody solution in 10% FCS/PBS buffer, overnight at 4 °C. On the next day, the embryos were washed and incubated with secondary antibodies, overnight at 4 °C. After that, the samples were washed and mounted on glass-bottom plates in PBS droplets under oil. Imaging was performed using Zeiss LSM780 system equipped with ZEN software (v2.0 and higher), Fiji software (v2.0 and higher) was used for image analysis. The list of primary and secondary antibodies is presented on Table S2.

### Generation of teratomas and immunohistochemistry

Scid mice were injected subcutaneously with a 100 µl suspension containing 5 million ESC in PBS. Solid tumours were isolated and fixed at 4 °C overnight in 4% PFA/PBS. The samples were dehydrated in an ethanol series (30%, 50%, 70% and 100% in PBS) for 2 h each, followed by two 10 min incubations in 100% xylene, and then transferred to paraffin. Similarly, foetal stage embryos were fixed overnight and dehydrated. Samples embedded into paraffin blocks were sectioned at 7 µm using an HM355S microtome (Thermo). Haematoxylin/eosin staining and immunohistochemistry were carried out as previously described^78,79^. Images were acquired using Nikon Eclipse Ti2 system.

### Generation of embryoid bodies

ESC differentiation using the EBs assay was performed as previously described^80^. The EBs were harvested and dissociated into single cells for Seahorse analysis 14 days after induction of differentiation.

### Quantitative PCR

RNA isolation was performed using RNeasy Mini Kit (Qiagen, 74106) according to the manufacturer’s instructions and reverse transcribed using MMLV-Reverse transcriptase (Applied Biosystems). Transcript levels were quantified using iTaq SYBR Green Supermix (Bio-Rad). Relative gene expression was calculated using delta-delta Ct method and normalized to relative GAPDH expression. Primer pair sequences (5´-3´): Lima1 (AGCCAGAGACGAGCGAAAAC and GCACCTATTTCCCAGTCATCCA) Oct4 (AGACCATGTTTCTGAAGTGCCCG and CGCCGGTTACAGAACCATACTCG), Nanog (AGGGTCTGCTACTGAGATGCTCTG and CAACCACTGGTTTTTCTGCCACCG), GAPDH (TGAAGCAGGCATCTGAGGG and CGAAGGTGGAAGAGTGGGAG).

### RNA sequencing and bioinformatic analysis

RNA was isolated from cells using the NucleoSpin RNA isolation kit following the manufacturer’s protocol (Macherery and Nagel). Samples with RNA integrity number (RIN) above 7 were processed further. The mRNA enrichment which was performed using NEBNext Poly(A) mRNA Magnetic Isolation Module and cDNA library was prepared using NEBNext Ultra II Directional RNA Library Prep Kit. Sequencing was performed on the NextSeq 500 system (75 cycles, high output, v2.5) in the Core Facility Genomics of the Medical Faculty, University of Münster.

Reads were trimmed with fastp (v0.20.0), setting the ‘-3’, ‘-cut_tail_window_size 1’, and ‘-cut_tail_mean_quality 20’ options. The trimmed RNA-seq data was analyzed with Salmon (v1.4.0)^81^ and DESeq2 (v1.30.0)^82^. Briefly, transcript level abundance for all samples was estimated using Salmon, with indices for the Mus musculus (Gencode v23) and Homo sapiens (Gencode v35) transcriptomes and options ‘-validateMappings’, ‘-seqBias’ and ‘-gcBias’. The resulting quantification data were imported into R (v4.0.3) with tximeta (v1.8.2)^83^ and summed up to the gene level. Differential expression per cell type between control/wild-type and Lima1 overexpression/knockdown was tested with DESeq2. Genes with an adjusted *p*-level < 0.01 were called as differentially expressed. Abundance levels for genes of special interest were z-score transformed and plotted with ComplexHeatmap (v2.6.2)^84^. GO gene set over-representation analysis was performed with clusterProfiler (v3.18.0)^85^, using the set of all genes with valid GO annotation as background, respectively. All non-heatmap plots were created using ggplot2 (v3.3.2).

### ChIP-seq and ATAC-seq data analysis

ChIP-seq dataset of Nanog^35^, Sox2^32^, Sall4^33^, Nr0b1^28^, Klf4^29^, Oct4^27^, Tcf3^35^, β-cat^34^ and Polr2a^31^ binding in ESC, as well as ESC ATAC-seq data^30^ were obtained from the Cistrome Data Browser^86,87^. Peak visualisation was performed using Integrated Genome Viewer software (v2.4.15).

### Protein extraction and immunoblotting

Cells were washed with PBS, scraped and pelleted by centrifugation at 16,000 × *g* for 10 min. For immunoblotting, the cell pellets were lysed in buffer consisting of 10 mM Tris-HCl pH 7.6, 150 mM NaCl, 2 mM MgCl_2_, 2 mM EDTA, 0.1% Triton X-100, 10% Glycerol and protease inhibitors (Sigma, 5892791001) for 15 min on ice. For mass spectrometry analysis, the cells were lysed in buffer consisting of 8 M Urea, 50 mM Tris-HCl pH 8.0, 150 mM NaCl, 1 mM EDTA and protease inhibitors for 15 min on ice. After that supernatants were collected by centrifugation at 16,000 × *g* for 15 min and the protein concentration was determined using BCA kit (Thermo, 23227) following manufacturer’s instructions. The protein samples were run on SDS-Polyacrylamide gel alongside and then transferred onto a PVDF membrane. After that the membrane was incubated in 5% dry milk or BSA in PBST (0.01% Tween-20/PBS) buffer, for 30 min at room temperature and then incubated with primary antibodies at 4 °C, overnight. On the next day, the membrane was washed with PBST and incubated with HRP-conjugated secondary antibodies for 45 min at room temperature. The proteins were detected using the ECL (GE Healthcare, GERPN3243) or ECL Prime (GE Healthcare, GERPN2232) kits by exposing the membranes to ECL Hyperfilms (GE Healthcare, GE28-9068-36). Scans of the immunoblots used in this study are presented in Fig. S9.

### Proximity biotinylation

APEX2 proximity biotinylation in ESC expressing full-length Lima1-HA-APEX2 construct was performed following previously published protocol^88^. Briefly, the cells were cultured in the presence of 500 μM biotin-phenol for 30 min, followed by pulse treatment with 1 mM H_2_O_2_ for 1 min. After extensive washing with quenching buffer (10 mM sodium ascorbate, 5 mM Trolox, 10 mM sodium azide in PBS), the cells were lysed and biotinylation was verified by immunoblotting (see Protein extraction and Immunoblotting) using the following modifications in the protocol: After transfer to PDVF membrane, the membrane was blocked with 5% skim milk in 0.2% Triton X-100/TBS (TBS-Triton) for 1 h, washed briefly three times with 0.05% Tween-20 in TBS (TBS-Tween) and incubated with streptavidin-HRP in 3% BSA in TBS-Triton for 1 h. Following extensive washing (three times in TBS-Tween, one time in 10% FCS/ 1% Triton X-100/TBS-Tween and five times in TBS-Tween), protein biotinylation was visualized using ECL kit, by exposing the membranes to ECL Hyperfilms.

Approximately 1 mg of protein lysate was pre-digested using endoproteinase LysC (1:100, w/w) for 2 h at 30 °C. Next, the concentration of urea was reduced to 2 M using 50 mM ammonium bicarbonate buffer (pH 8.0) and the digestion was extended overnight at 37 °C by addition of trypsin (1:50, w/w). The digestion was stopped by the addition of trifluoroacetic acid (TFA) to a final concentration of 1%, desalted using C18 Sep-Pak cartridges (Waters) and lyophilized. Lyophilizates were dissolved in 1.5 ml IAP buffer (10 mM sodium phosphate dibasic, 50 mM MOPS pH 7.2, 50 mM NaCl). The pH was adjusted to seven with 1 M Tris. The pull-down was performed following previously published protocol^89^. In brief, 20 μl of agarose beads covalently linked to biotin antibody (ImmuneChem, ICP0615) were first washed with IAP buffer. After that, the beads were incubated with the sample at 4 °C for 1 h on a rotator. Following four times washing with PBS, the bound peptides were eluted using 0.15% (v/v) TFA, desalted using C18 Stage tips and stored on-tip until subjected to measurement on the MS system.

### Mass spectrometry

Peptide samples in 0.1% formic acid were measured on a hybrid TIMS-quadrupole time of flight mass spectrometer (timsTOF pro) coupled to a nanoElute UHPLC system via a Captive Spray ion source (Bruker). Peptides were separated on the nanoElute within 90 min with a linear gradient from 3–35% buffer B on a self-packed C18 reverse phase capillary column with pulled emitter tip (nanoseparations; 360 µm OD x 75 µm ID × 250 mm; Reprosil pur C18-aq, 1.9 µm; Dr Maisch) using a constant flow of 300 nl/min (Buffer A: 0.1% formic acid; Buffer B: 0.1% formic acid in acetonitrile). At the end of the gradient, the column was flushed with 90% B before re-equilibration at starting conditions. MS and MS/MS spectra were recorded in positive mode from m/z 100 to 1700 Da, using the PASEF scan mode. Each duty cycle consisted of 1 TIMS-MS and an average of ten PASEF MS/MS frames, each one containing multiple MS/MS spectra, which resulted in a total cycle time of 1.1 s. To exclude the majority of singly charged ions with low m/z for PASEF precursor selection, a polygon filtering was applied to the m/z over ion mobility area. For the 90 min runs target intensity was set to 20,000 cts/s and an ion mobility range (1/K0) of 0.6–1.6 Vs/cm^2^ was used. Data were acquired with a 100 ms ramp time. The Bruker Hystar/oTOF Control software was used to control the LC-MS system and record the data (version 3.2; Bruker Daltonics).

### Mass spectrometry data analysis and label-free quantification

MS files were processed using the MaxQuant computational platform (version 1.6.14.0)^90^. Identification of peptides and proteins was enabled by the built-in Andromeda search engine by querying the concatenated forward and reverse mouse Uniprot database (UP000005640_9606.fasta; version April 2019) including common lab contaminants. Default values of MaxQuant remained unchanged. Trypsin was selected as a protease allowing up to two missed cleavages, and the peptide search was limited to a minimum length of 7 amino acids and a maximum mass of 4600 Da. Oxidation of methionine, protein N-terminal acetylation, deamidation as well as the biotinylation of lysine (+226.07759 Da), biotin-phenol modification of tyrosine (+361.14601 Da), and oxidized-biotin-phenol modification of tyrosine (+377.141 Da) were set as variable modifications, while carbamidomethylations of cysteine were defined as fixed modification. For peptide and protein identifications, a minimum false discovery rate (FDR) of 1% was required. The match between runs option was enabled setting a retention time matching window of 0.7 min that included also a 1/K0 matching window of 0.05 V ∙ s/cm^2^ for measurements that were obtained on the timsTOF. The calculation of iBAQ values was enabled on all occasions.

Relative label-free quantification using the MaxQuant LFQ algorithm (version 1.6.14.0) was based on the measurements of three biological replicates for each sample. First, reverse and contaminant hits as well as proteins that were identified by a single modified peptide only were eliminated from the list of identified protein groups. Proteins eventually included for further analysis had to be identified with at least one unique peptide.

Following the labelling reaction and subsequent sample preparation steps, biotin-phenol containing peptides were specifically enriched. Matching back those labelled peptides to the proteins from which they were derived, indicated that these proteins must have been present in close proximity to the APEX enzyme. In contrast, control samples that were incubated without the biotin-phenol substrate were completely void of those modified peptides. Thus, while we formally cannot exclude the possibility that also more abundant proteins were accidentally present in the vicinity of Lima1 during the short labelling reaction and could have been labelled by the APEX activity, we consider that primarily static, as well as more dynamically binding interactors of Lima1, were predominantly labelled.

### TMRE assay

The cells were incubated in culture medium supplemented with 300 nM MitoTracker Green (Invitrogen, M7514) for 30 min. After that the cells were washed twice with PBS and once with culture medium and then incubated in medium supplemented with 15 nM TMRE (Biomol, ABD-22220) for 30 min. The negative controls were incubated additionally with 20 µM of the oxidative phosphorylation uncoupler carbonyl cyanide m-chlorophenylhydrazone (CCCP; Sigma, C2759) for 10 min. After that cells were washed, harvested and subjected to FACS analysis. DAPI was used to select the live cells. The TMRE signal intensity was normalized to MitoTracker Green signal.

### Pyruvate kinase activity assay

Photometric analysis of pyruvate kinase activity in ESC lysates was performed using a pyruvate kinase activity kit (Sigma, MAK072), following the manufacturer’s instruction.

### ROS assay

Analysis of ROS levels was performed using ROS detection assay kit (PromoCell, PK-CA577-K936-1) according to the manufacturer’s instructions.

### Mitochondrial stress test assay

The OCR was measured with the Seahorse XFe96 Extracellular Flux Analyzer. The cells were seeded on 96-well XF cell culture microplates 24 h prior to analysis. Fresh medium was supplied before starting the OCR analysis. The assay was performed according to the manufacturer’s instructions using 0.5 µM Oligomycin, 1 µM FCCP and 0.5 µM Rotenone/Antimycin A. After the OCR measurement, the cell number per well was determined by labelling the nuclei with Hoechst 33342.

### Annexin V assay

ESCs were individualized by trypsinization, whereas hiPSCs were individualized by Accutase treatment for 10 min at 37 °C. The cells were then resuspended and cultured on cell-repellent plates for 2 h at 37 °C. After that, the cell death was determined by FACS analysis using Annexin V Alexa Fluor 568 conjugate kit (Thermo, A13202), according to the manufacturer’s instructions. DAPI was added just before the FACS analysis.

### Ultrastructural analysis

Cells were initially fixed in 2% paraformaldehyde, 0.2% glutaraldehyde in 0.1 M phosphate buffer, pH 7.2, and further processed for cryo immunogold labelling as previously described^91^. From the resulting frozen sample blocks, 50 nm ultrathin sections were cut at −110 °C. The sections were retrieved in a mixture of sucrose/methylcellulose and transferred on formvar coated copper grids (200-mesh, hexagonal). Single immunogold labelling was performed against HA-tagged Lima1 with an antibody recognizing HA-tag (mouse, clone 16.B12, Biolegend), detected by a bridging antibody, which was recognized by 10 nm protein A gold conjugate (CMC, Utrecht, Netherlands).

For double immunogold labelling antibodies against actin (mouse, 4F7, kind gift from Professor Brigitte Jockusch and Sabine Buchmeier, Antibody Facility TU-Braunschweig) and Lima1 (rabbit, A300-102A, Bethyl Laboratories, Inc.) were used stepwise, detected by 15 nm and 10 nm protein A gold, respectively. The samples were analyzed at 80 kV using a transmission electron microscope (Tecani12-Biotwin, Thermo Fisher Scientific). Representative images were exposed on ditabis imaging plates (Ditabis, Pforzheim, Germany).

### Quantification and statistical analysis

All statistical analyses were performed using GraphPad Prism unless otherwise stated. Information about number of independent repetitions, sample size and statistical tests are indicated in the figure legends.

### Reporting summary

Further information on research design is available in the Nature Research Reporting Summary linked to this article.

## Supplementary information

Supplementary Information

Description of Additional Supplementary Files

Supplementary Dataset 1

Supplementary Dataset 2

Supplementary Dataset 3

Supplementary Dataset 4

Supplementary Movie 1

Supplementary Movie 2

Reporting Summary

## Data Availability

The RNA-seq data generated in this study have been deposited in the ArrayExpress database under accession code “E-MTAB-10301”. The proteomics data data data generated in this study have been deposited in the ProteomeXchange database under accession code “ PXSD025293”. Uniprot database UP000005640_9606.fasta; version April 2019 used for Mass Spectrometry analysis is available at “Uniprot [ftp://ftp.uniprot.org/pub/databases/uniprot/previous_releases/release-2019_04/knowledgebase/]”. All other relevant data supporting the key findings of this study are available within the article and its Supplementary Information files or from the corresponding author upon reasonable request. Source data are provided with this paper.

## Source data

Source Data
