# Supplementary material for: Lima1 mediates the pluripotency control of membrane dynamics and cellular metabolism
Source: Nat Commun. 2022 Feb 1;13:610. doi: 10.1038/s41467-022-28139-5 (PMC8807836; doi:10.1038/s41467-022-28139-5)
Supplement: Supplementary file 1 — Supplementary Information [file 41467_2022_28139_MOESM1_ESM.pdf]

Supplementary Information

Lima1 mediates the pluripotency control of membrane dynamics and cellular metabolism

Duethorn et al

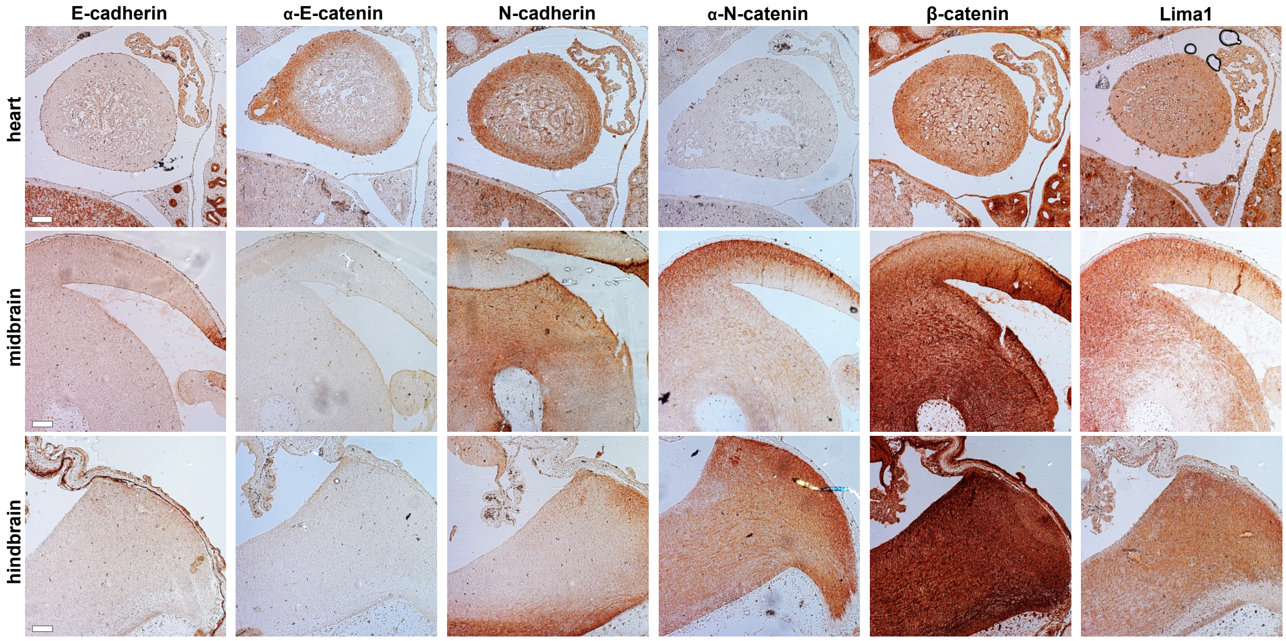

Figure S1. Lima1 expression in E14.5 embryos.
Heart and brain regions of E14.5 embryos stained for E-cad, α-E-cat, N-cad, α-N-cat, β-cat or Lima1.
Scale bar, 100 μm. Experiments were repeated independently at least three times with similar results. Related to Figure 1.

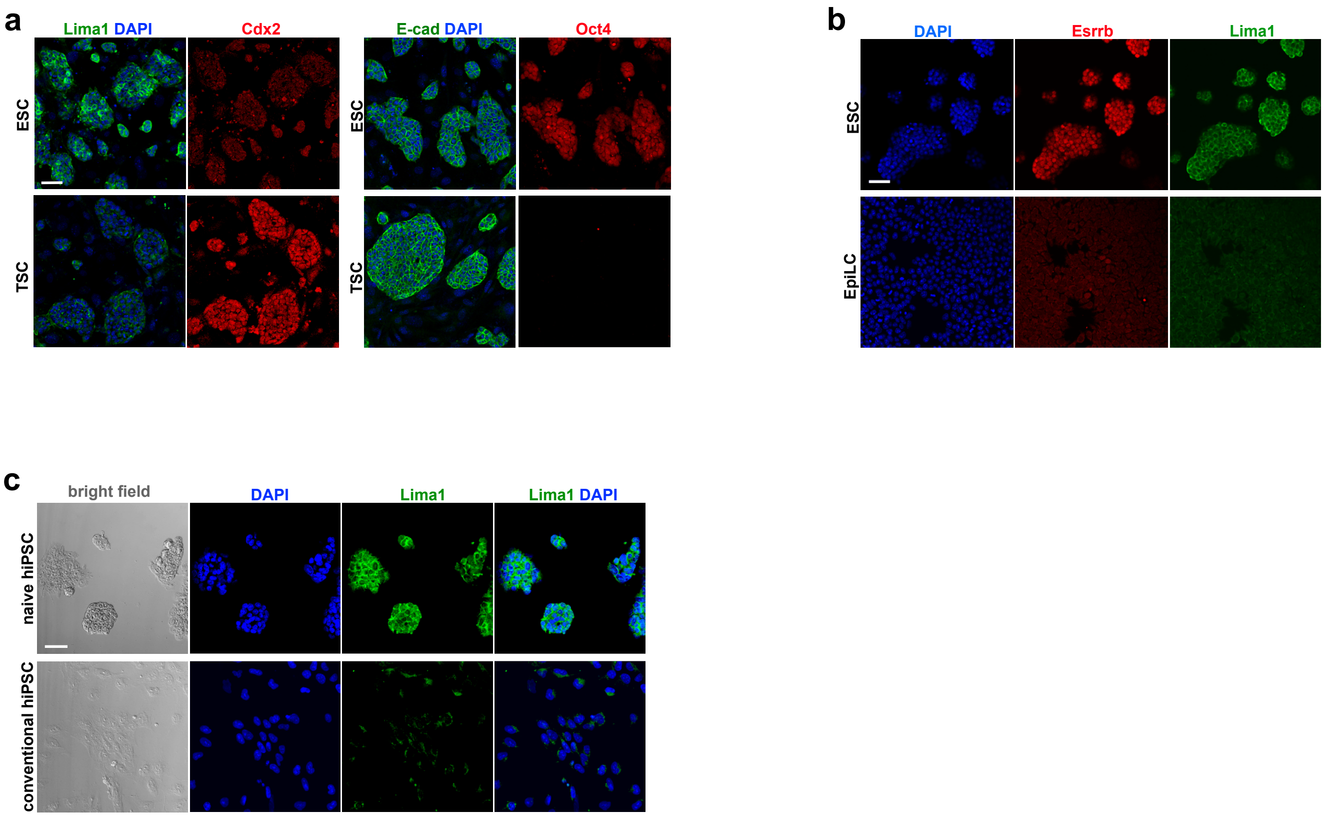

Figure S2. Lima1 expression in pluripotent stem cells.
a) ESC and TSC stained for Lima1 and Cdx2 (left panel) or E-cad and Oct4 (right panel).
b) ESC and EpiLC stained for Esrrb and Lima1.
c) Naïve hiPSC and conventional hiPSC stained for Lima1.
Scale bars, (a), (b), (c), 50 μm. Experiments were repeated independently at least three times (a) or two times (b, c) with similar results.
Related to Figure 2.

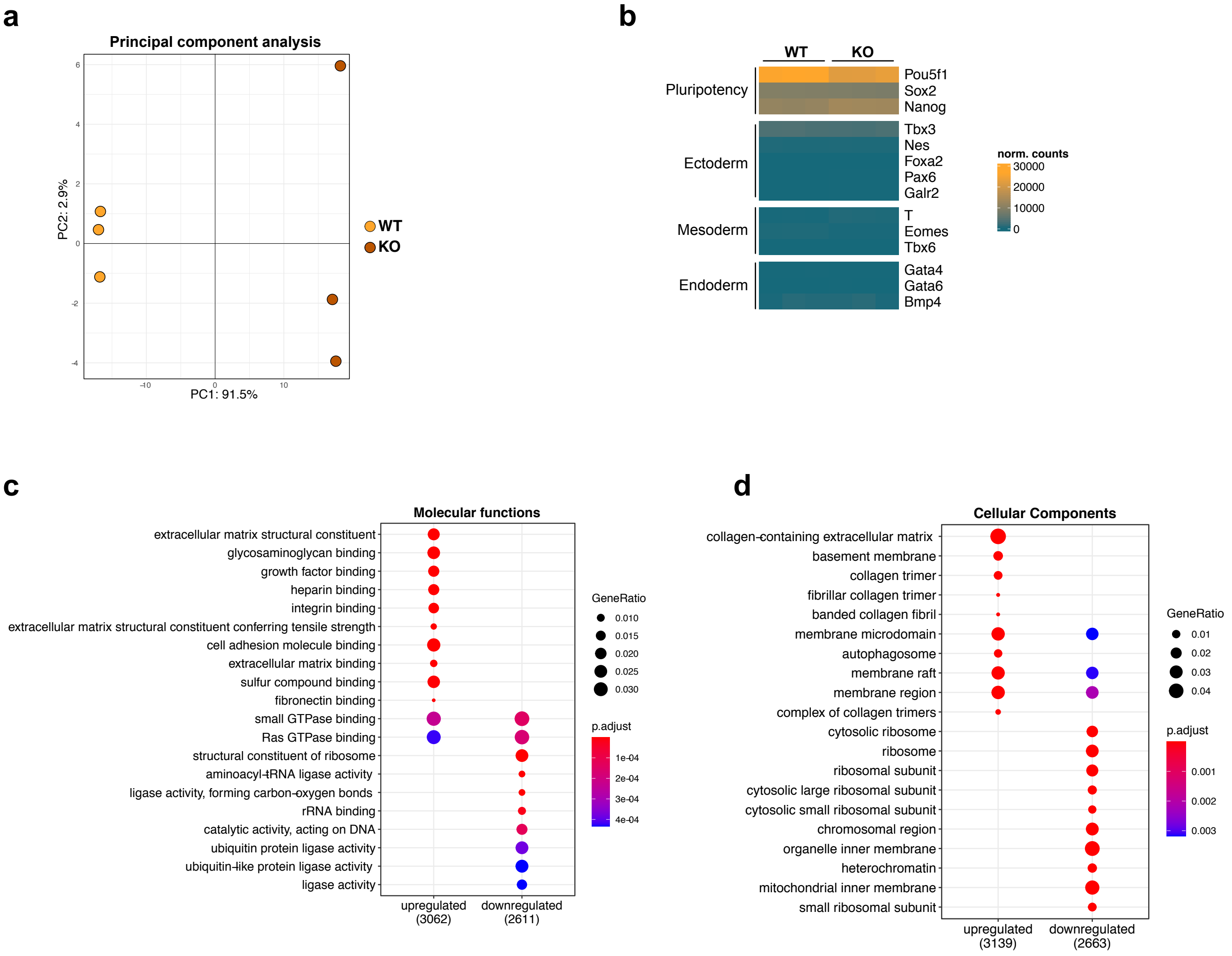

Figure S3. RNA-seq analysis of Lima1 deletion in ESC.
a) Principal component analysis of WT and Lima1 KO ESC transcriptomes.
b) Expression of pluripotency and lineage differentiation markers in WT and Lima1 KO ESC.
c) Gene ontology (GO) enrichment analysis “Molecular functions”.
d) Gene ontology (GO) enrichment analysis “Cellular Components”.
Related to Figure 3.

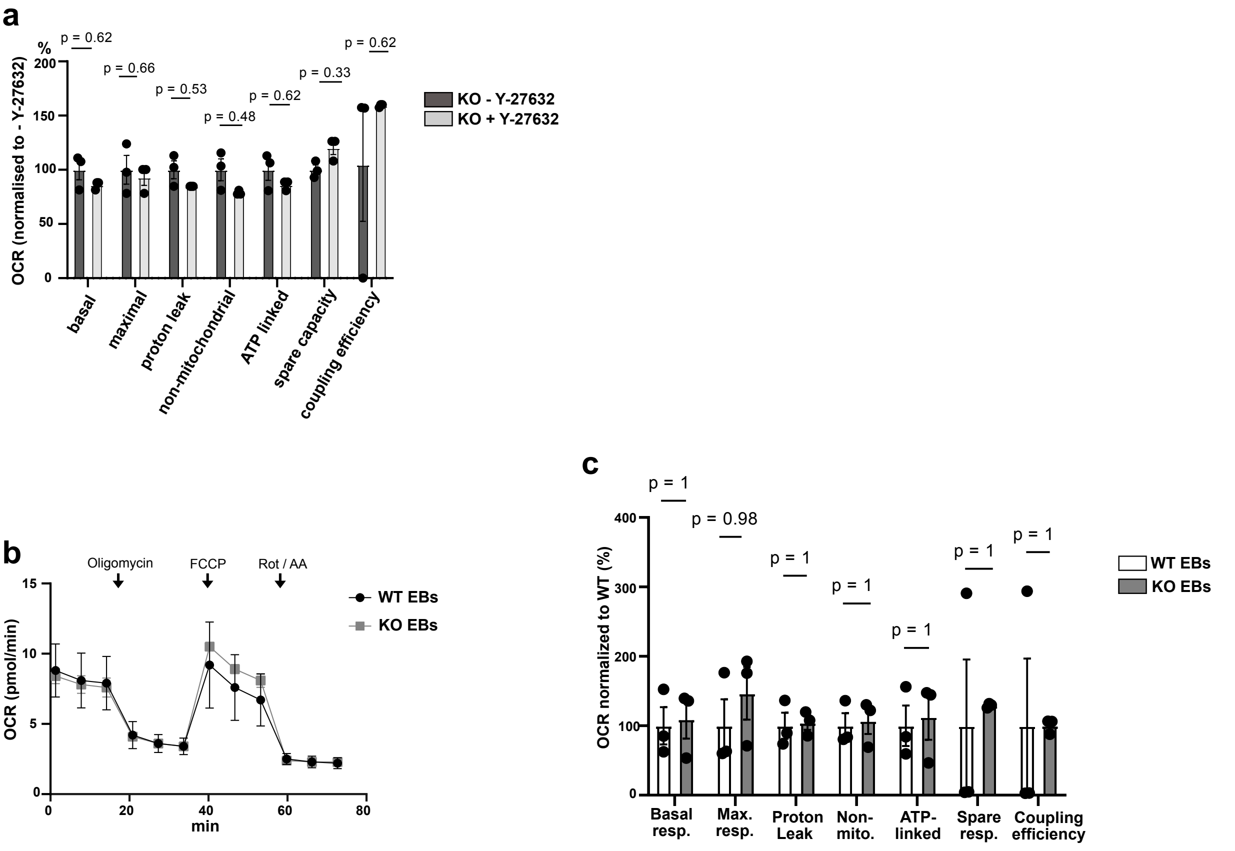

Figure S4. Seahorse analysis of Lima1 KO ESC and EBs.
a) Quantification of OCR in Lima1 KO ESC treated or untreated with Y-27632. Data represent mean values ± SEM from n = three independent experiments, with 10 measurements per genotype, unpaired Student's t-test, 2-sided.
b) OCR measurement in Lima1 KO or WT EBs using the Seahorse mitochondrial stress test assay. FCCP - Carbonyl cyanide-4 (trifluoromethoxy) phenylhydrazone; Rot – Rotenone; AA – Antimycin. Three independent experiments, mean values ± SEM.
c) Quantification of the OCR in Lima1 KO or WT EBs. Data represent mean values ± SEM with n = three independent experiments, with 10 measurements per genotype, unpaired Student's t-test, 2-sided.
Related to Figure 5.

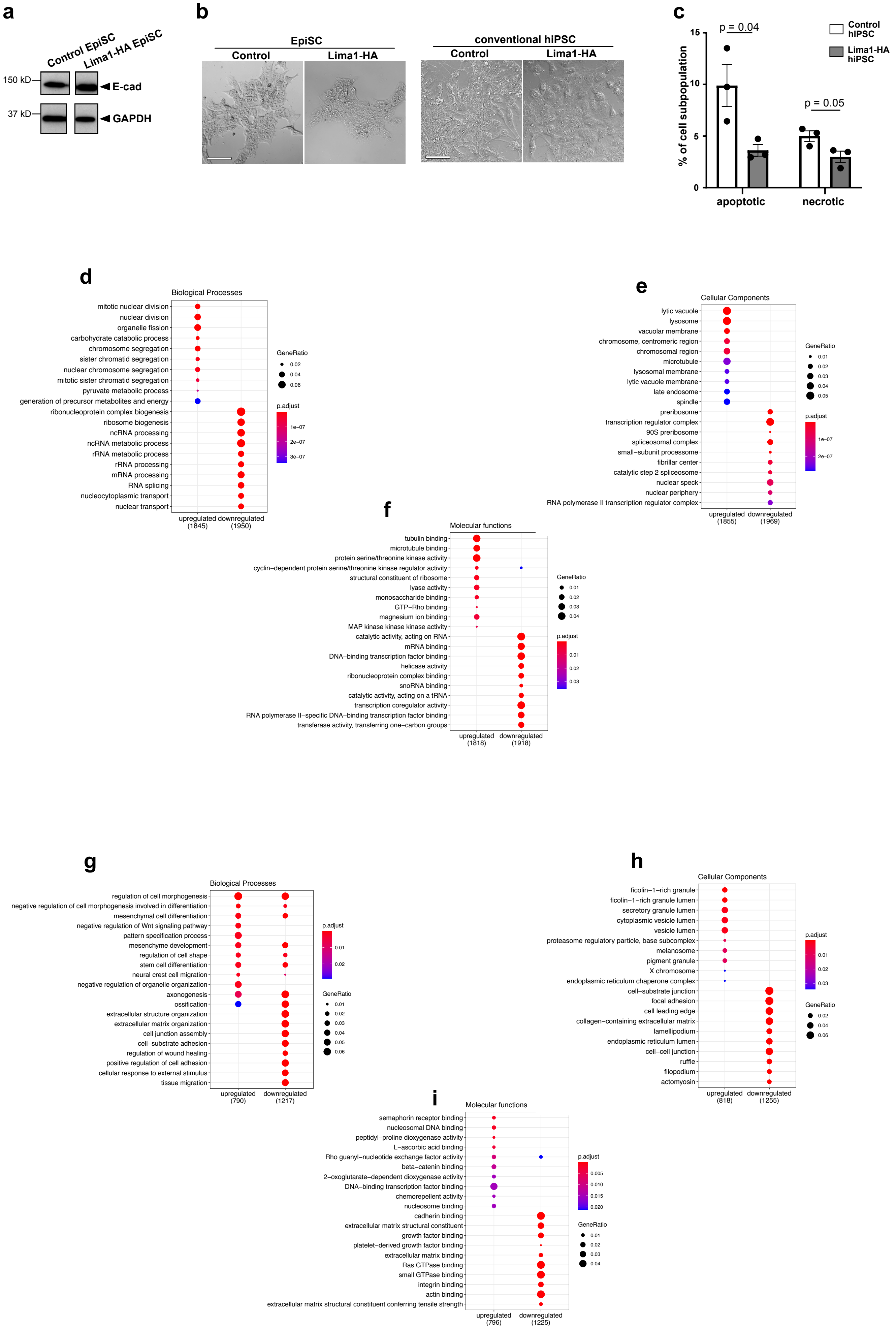
a
Control EpiSC
Lima1-HA EpiSC
150 kD
E-cad
37 kD
GAPDH
b
EpiSC
Control
Lima1-HA
conventional hiPSC
Control
Lima1-HA
c
p = 0.04
p = 0.05
% of cell subpopulation
15
10
5
0
apoptotic
necrotic
Control hiPSC
Lima1-HA hiPSC
d
Biological Processes
mitotic nuclear division
nuclear division
organelle fission
carbohydrate catabolic process
chromosome segregation
sister chromatid segregation
nuclear chromosome segregation
mitotic sister chromatid segregation
pyruvate metabolic process
generation of precursor metabolites and energy
ribonucleoprotein complex biogenesis
ribosome biogenesis
ncRNA processing
ncRNA metabolic process
rRNA metabolic process
rRNA processing
mRNA processing
RNA splicing
nucleocytoplasmic transport
nuclear transport
upregulated (1845)
downregulated (1950)
GeneRatio
0.02
0.04
0.06
p.adjust
1e−07
2e−07
3e−07
e
Cellular Components
lytic vacuole
lysosome
vacuolar membrane
chromosome, centromeric region
chromosomal region
microtubule
lysosomal membrane
lytic vacuole membrane
late endosome
spindle
preribosome
transcription regulator complex
90S preribosome
spliceosomal complex
small−subunit processome
fibrillar center
catalytic step 2 spliceosome
nuclear speck
nuclear periphery
RNA polymerase II transcription regulator complex
upregulated (1855)
downregulated (1969)
GeneRatio
0.01
0.02
0.03
0.04
0.05
p.adjust
1e−07
2e−07
f
Molecular functions
tubulin binding
microtubule binding
protein serine/threonine kinase activity
cyclin−dependent protein serine/threonine kinase regulator activity
structural constituent of ribosome
lyase activity
monosaccharide binding
GTP−Rho binding
magnesium ion binding
MAP kinase kinase kinase activity
catalytic activity, acting on RNA
mRNA binding
DNA−binding transcription factor binding
helicase activity
ribonucleoprotein complex binding
snoRNA binding
catalytic activity, acting on a tRNA
transcription coregulator activity
RNA polymerase II−specific DNA−binding transcription factor binding
transferase activity, transferring one−carbon groups
upregulated (1818)
downregulated (1918)
GeneRatio
0.01
0.02
0.03
0.04
p.adjust
0.01
0.02
0.03
g
Biological Processes
regulation of cell morphogenesis
negative regulation of cell morphogenesis involved in differentiation
mesenchymal cell differentiation
negative regulation of Wnt signaling pathway
pattern specification process
mesenchyme development
regulation of cell shape
stem cell differentiation
neural crest cell migration
negative regulation of organelle organization
axonogenesis
ossification
extracellular structure organization
extracellular matrix organization
cell junction assembly
cell−substrate adhesion
regulation of wound healing
positive regulation of cell adhesion
cellular response to external stimulus
tissue migration
upregulated (790)
downregulated (1217)
p.adjust
0.01
0.02
GeneRatio
0.01
0.02
0.03
0.04
0.05
0.06
h
Cellular Components
ficolin−1−rich granule
ficolin−1−rich granule lumen
secretory granule lumen
cytoplasmic vesicle lumen
vesicle lumen
proteasome regulatory particle, base subcomplex
melanosome
pigment granule
X chromosome
endoplasmic reticulum chaperone complex
cell−substrate junction
focal adhesion
cell leading edge
collagen−containing extracellular matrix
lamellipodium
endoplasmic reticulum lumen
cell−cell junction
ruffle
filopodium
actomyosin
upregulated (818)
downregulated (1255)
p.adjust
0.01
0.02
0.03
GeneRatio
0.02
0.04
0.06
i
Molecular functions
semaphorin receptor binding
nucleosomal DNA binding
peptidyl−proline dioxygenase activity
L−ascorbic acid binding
Rho guanyl−nucleotide exchange factor activity
beta−catenin binding
2−oxoglutarate−dependent dioxygenase activity
DNA−binding transcription factor binding
chemorepellent activity
nucleosome binding
cadherin binding
extracellular matrix structural constituent
growth factor binding
platelet−derived growth factor binding
extracellular matrix binding
Ras GTPase binding
small GTPase binding
integrin binding
actin binding
extracellular matrix structural constituent conferring tensile strength
upregulated (796)
downregulated (1225)
p.adjust
0.005
0.010
0.015
0.020
GeneRatio
0.01
0.02
0.03
0.04

Figure S5. Ectopic expression of Lima1 in primed pluripotent stem cells.
a) Western blot analysis of E-cad expression in control and Lima1-HA EpiSC.
b) Control and Lima1-HA EpiSC colonies (left panel), control and Lima1-HA hiPSC colonies (right panel).
c) Proportion of apoptotic and necrotic control and Lima1-HA hiPSC. The cells were individualized using accutase and cultured on cell-repellent plates for 2 h at 37 °C. After that, the cell death was determined by annexin V assay in combination with DAPI, three independent experiments, data represent mean ± SEM, unpaired Student’s t-test, 2-sided.
d) GO enrichment analysis “Biological processes” of control and Lima1-HA-expressing EpiSC.
e) GO enrichment analysis “Cellular Components” of control and Lima1-HA expressing-EpiSC.
f) GO enrichment analysis “Molecular functions” of control and Lima1-HA-expressing EpiSC.
g) GO enrichment analysis “Biological processes” of control and Lima1-HA-expressing conventional hiPSC.
h) GO enrichment analysis “Cellular Components” of control and Lima1-HA-expressing conventional hiPSC.
i) GO enrichment analysis “Molecular functions” of control and Lima1-HA-expressing conventional hiPSC.
Scale bar, (b), 50 µm. Experiments were repeated independently two times (a, b) with similar results.
Related to Figure 6.

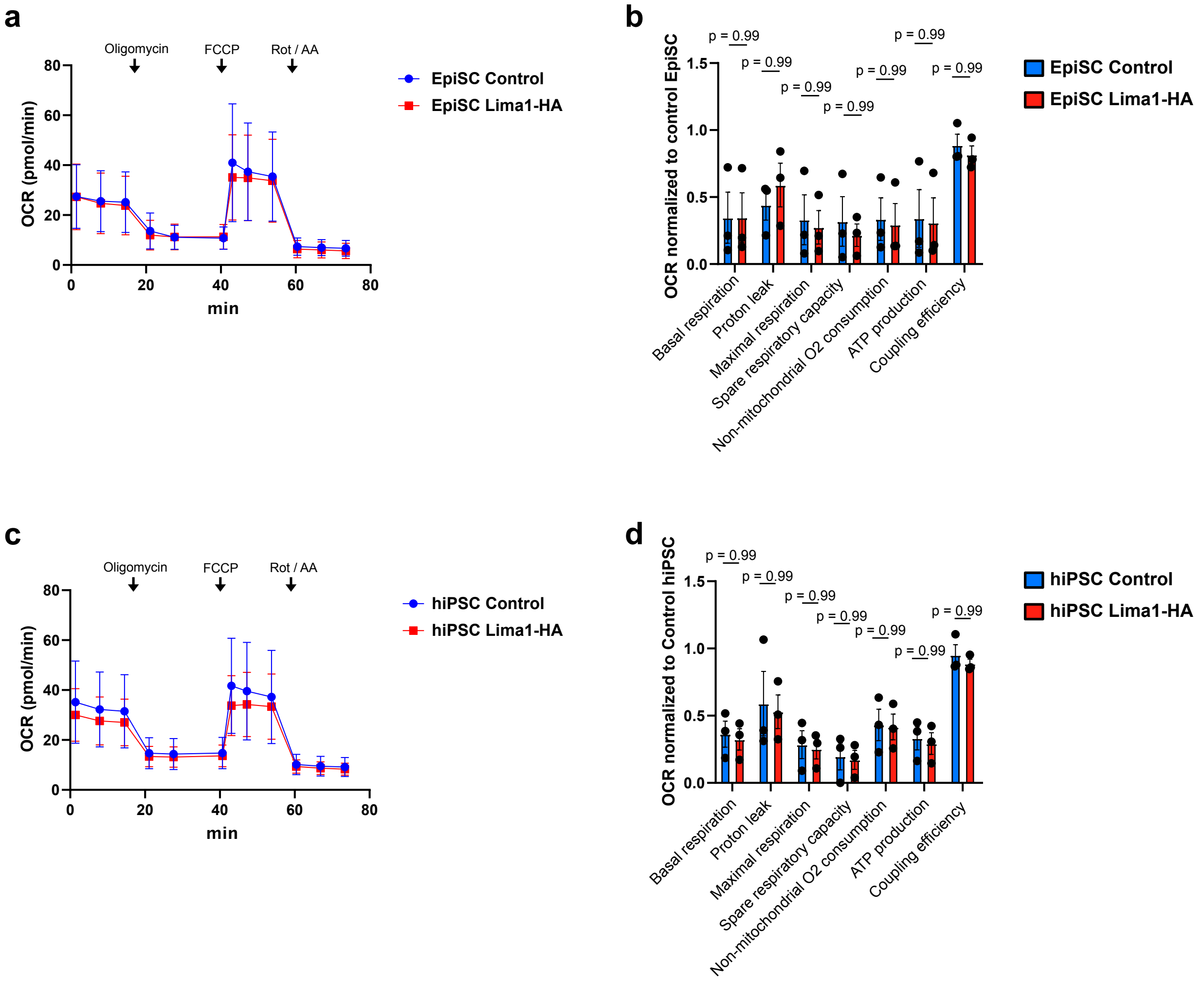

Figure S6. Seahorse mitochondrial stress test assay in EpiSC and hiPSC.
a) OCR measurement using the Seahorse mitochondrial stress test assay in control and Lima1-HA EpiSC. FCCP - Carbonyl cyanide-4 (trifluoromethoxy) phenylhydrazone; Rot – Rotenone; AA – Antimycin. Three independent experiments, mean values ± SEM.
b) Quantification of the OCR analysis in control and Lima1-HA EpiSC, three independent experiments. Data represent mean ± SEM, unpaired Student's t-test, 2-sided.
c) OCR measurement using the Seahorse mitochondrial stress test assay in control and Lima1-HA hiPSC. FCCP - Carbonyl cyanide-4 (trifluoromethoxy) phenylhydrazone; Rot – Rotenone; AA – Antimycin. Three independent experiments, mean values ± SEM.
d) Quantification of the OCR analysis in control and Lima1-HA hiPSC, three independent experiments. Data represent mean ± SEM, unpaired Student's t-test, 2-sided.
Related to Figure 7.

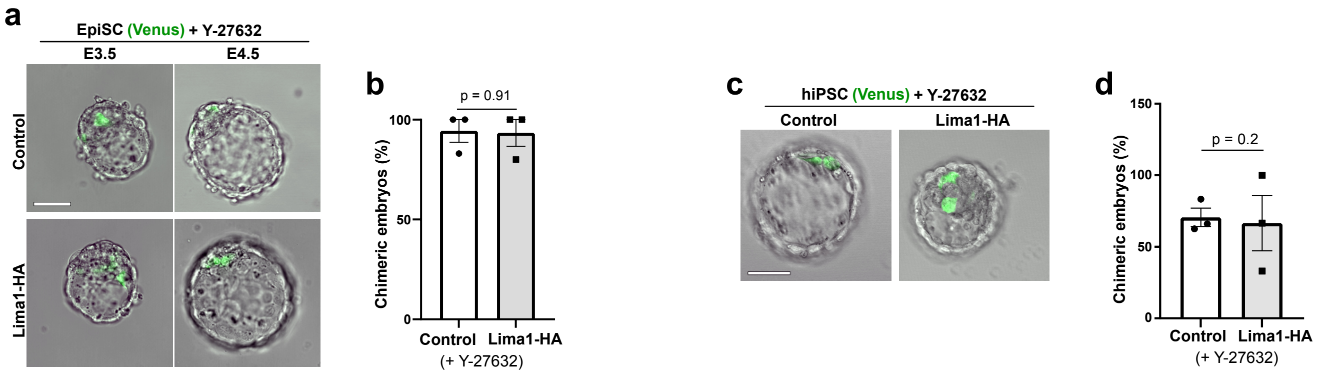

Figure S7. Engraftment of primed pluripotent cell in mouse pre-implantation embryos upon treatment with Y-27632.
a) E3.5 and E4.5 chimeric blastocysts generated via morula aggregation using control or Lima1-HA-expressing EpiSC and treated with Y-27632.
b) Quantification of the chimeric blastocysts containing control (embryos) or Lima1-HA-expressing (embryos) EpiSC at E4.5. Data represent mean values ± SEM with n = three independent experiments, unpaired Student's t-test, 2-sided.
c) Chimeric blastocysts generated via morula aggregation using control or Lima1-HA-expressing conventional hiPSC and treated with Y-27632.
d) Quantification of the chimeric blastocysts containing control (20 embryos) or Lima1-HA-expressing (14 embryos) conventional hiPSC at E4.5. Data represent mean values ± SEM with n = three independent experiments, unpaired Student's t-test, 2-sided.
Scale bars, (a), (c), 20 μm. Related to Figure 8.

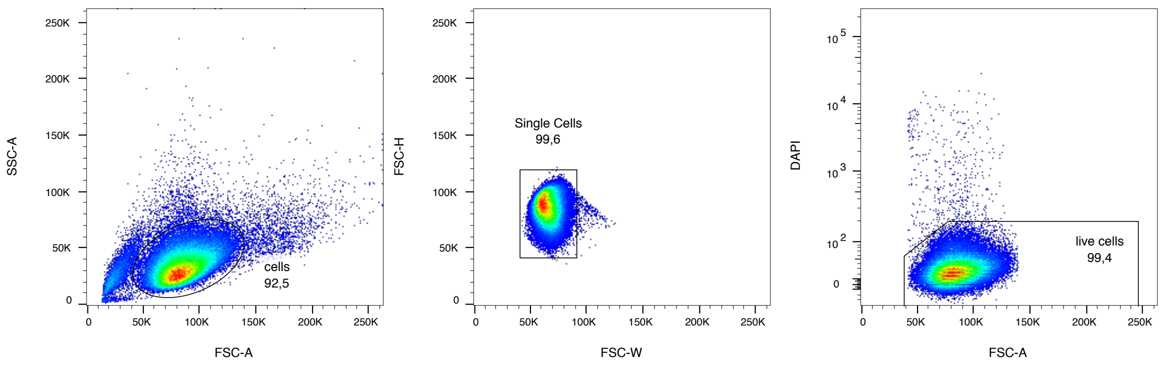

Figure S8. FACS gating.
Single viable cells were selected based on FSC- and SSC-gating. DAPI was used to select the live cells.

FIG 2A

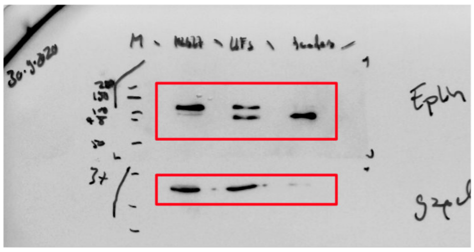

FIG 2D

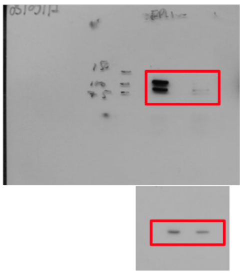

FIG 2E

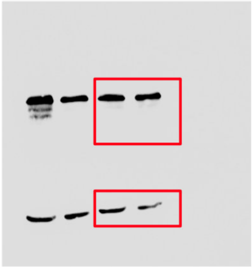

FIG 2H

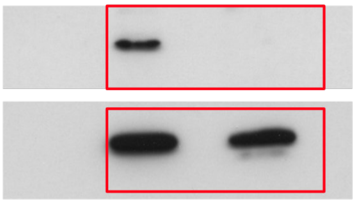

FIG 2i

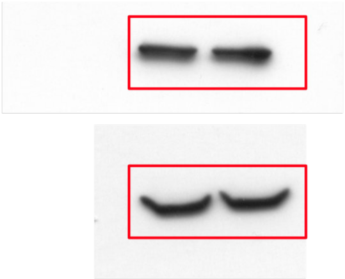

FIG 2J

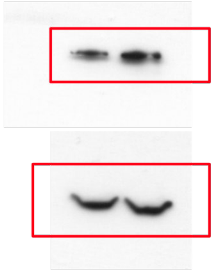

FIG 3B

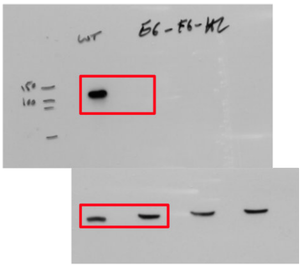

FIG 3I

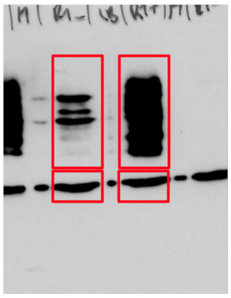

FIG 6B

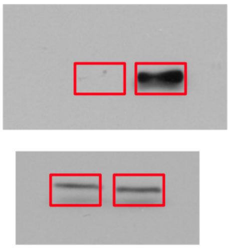

FIG 6D

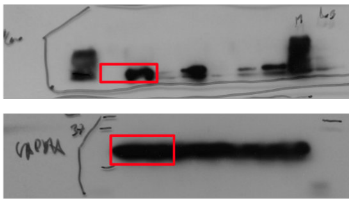

FIG 7D

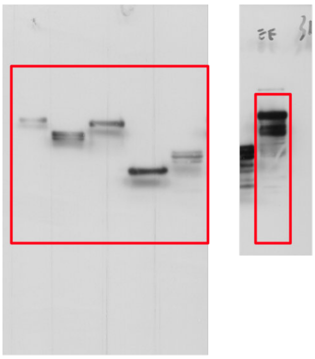

FIG S5A

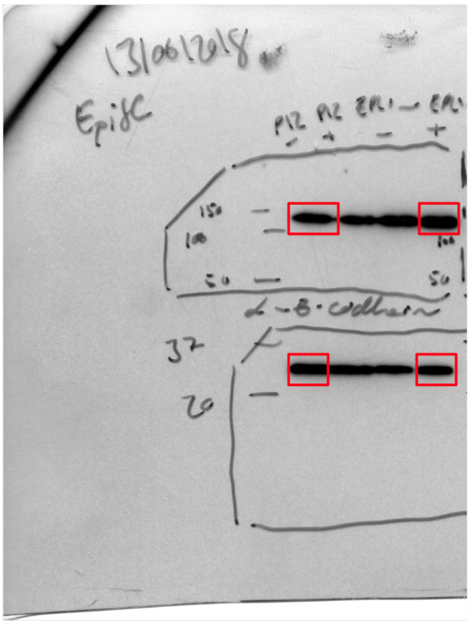

Figure S9. Scans of the immunoblots used in this study.
Red squares indicate the area of the blots used in the figures.

Table S1. Publicly available ChIP-seq datasets used in this study.

Reference	27	28	29	30	31	32	33	34	35
Factor	Oct4	Nr0b1	Klf4	ATAC-seq	Polr2a	Sox2	Sall4	B-catenin	Nanog, Tcf3
Culture conditions	N2B27 2i/LIF	Serum /LIF	Serum /LIF + feeders	Serum /LIF + feeders	N2B27 2i/LIF	Serum /LIF + feeders (feeders were removed before performing the ChIP-seq)	1 % Serum + 10 % KSR (gelatine coated)	Serum /2i/LIF	Serum /LIF (gelatine coated)

Table S2. Primary and secondary antibodies used in this study.

Description	Manufacturer	Cat. Number	Application and dilution
**Primary antibodies**			
b-actin	Sigma	A5316	ICC (1:200)
a-E-catenin	Cell Signaling	3236S	ICC (1:200), WB (1:1000)
a-E-catenin	Thermo	13-9700	ICC (1:200)
a-N-catenin	Cell Signaling	CD664	ICC (1:100)
b-catenin	BD Biosciences	610154	ICC (1:100), WB (1:1000)
Biotin antibody agarose	ImmuneChem	ICP0615	Pull-down (100 µg per 1 mg dried peptide)
Cdx2	Biogenex	MU392A-UC	ICC (1:200)
E-cadherin	BD Biosciences	610182	ICC (1:200), WB (1:1000)
Eomes/Tbr2	Abcam	AB23345	ICC (1:200)
Eplin	Proteintech	16639-1-AP	WB (1:500)
Eplin	Bethyl	A300-103A-M	ICC (1:100), WB (1:1000)
Eplin	Abe and Takeshi, 2008	[7]	ICC (1:200)
Esrrb	R&D	PP-H6705-00	ICC (1:200)
Gapdh	Cell Signaling	5174S	WB (1:2000)
Gata3	Cell Signaling	5852S	ICC (1:200)
GFP	R&D	AF4240	ICC (1:200)
HA-tag	Cell Signaling	3724S	ICC (1:500), WB (1:1000)
Nanog	Abcam	ab80892	ICC (1:200)
N-cadherin	BD Biosciences	610920	ICC (1:100)
Oct4	Santa Cruz	sc-5279	ICC (1:200)
Oct4	Cell Signaling	83932S	ICC (1:200)
RFP	Biomol	600-401-379	ICC (1:200)
Sox2	Calbiochem	246510	ICC (1:200)
Sox2	Cell Signaling	23064S	ICC (1:200)
Phospho-ERM	Cell Signaling	3726S	ICC (1:200)
Podocalyxin	R&D	MAB1556	ICC (1:100)
Troma-1	Kemler et al, 1981	[92]	ICC (1:200)
**Secondary Antibodies**			
Peroxidase AffiniPure Goat Anti-Mouse IgG + IgM (H+L)	Jackson ImmunoResearch	AB_2338451	WB (1:2000)
Phalloidin AF647	Cell Signaling	8940S	ICC (1:200)
Pierce High Sensitivity Streptavidin-HRP	Thermo	21130	WB (1:30000)
Rabbit IgG HRP Linked	GE Healthcare	NA934	WB (1:20000)
Secondary Donkey anti-mouse AF 488	Invitrogen	A-21202	ICC (1:200)
Secondary Donkey anti-mouse AF 594	Invitrogen	A-21203	ICC (1:200)
Secondary Donkey anti-mouse AF 647	Invitrogen	A-31571	ICC (1:200)
Secondary Donkey anti-goat AF 488	Invitrogen	A-11055	ICC (1:200)
Secondary Donkey anti-rabbit AF 488	Invitrogen	A-21206	ICC (1:200)
Secondary Donkey anti-rabbit AF 594	Invitrogen	A-21207	ICC (1:200)
Secondary Donkey anti-rat AF 647	Invitrogen	A21247	ICC (1:200)
