# Supplementary material for: Lima1 mediates the pluripotency control of membrane dynamics and cellular metabolism
Source: Nat Commun. 2022 Feb 1;13:610. doi: 10.1038/s41467-022-28139-5 (PMC8807836; doi:10.1038/s41467-022-28139-5)
Supplement: Supplementary file 2 — Description of Additional Supplementary Files [file 41467_2022_28139_MOESM2_ESM.docx]

Description of Additional Supplementary Files

Title: Supplementary Dataset 1.

Description: Gene expression levels in Lima1 KO ESC relative to WT ESC.

Title: Supplementary Dataset 2.

Description: Biotinylated factors identified in Lima1-APEX2 interactome analysis.

Title: Supplementary Dataset 3.

Description: Gene expression levels in Lima1-HA EpiSC relative to control EpiSC.

Title: Supplementary Dataset 4.

Description: Gene expression levels in Lima1- HA hiPSC relative to control hiPSC.

Title: Supplementary Movie 1.

Description: Time lapse recording of individualised WT ESC. Related to Figure 3.

Title: Supplementary Movie 2.

Description: Time lapse recording of Lima1 KO ESC, arrowheads indicate cells exhibiting membrane blebs. Related to Figure 3.
