# Supplementary material for: Lima1 mediates the pluripotency control of membrane dynamics and cellular metabolism
Source: Nat Commun. 2022 Feb 1;13:610. doi: 10.1038/s41467-022-28139-5 (PMC8807836; doi:10.1038/s41467-022-28139-5)
Supplement: Supplementary file 9 — Reporting Summary [file 41467_2022_28139_MOESM9_ESM.pdf]

nature portfolio

Corresponding author(s): Ivan Bedzhov

Last updated by author(s): Dec 20, 2021

Reporting Summary

Nature Portfolio wishes to improve the reproducibility of the work that we publish. This form provides structure for consistency and transparency in reporting. For further information on Nature Portfolio policies, see our Editorial Policies and the Editorial Policy Checklist.

Statistics

For all statistical analyses, confirm that the following items are present in the figure legend, table legend, main text, or Methods section.

n/a	Confirmed	
☐	☒	The exact sample size (*n*) for each experimental group/condition, given as a discrete number and unit of measurement
☐	☒	A statement on whether measurements were taken from distinct samples or whether the same sample was measured repeatedly
☐	☒	The statistical test(s) used AND whether they are one- or two-sided<br>*Only common tests should be described solely by name; describe more complex techniques in the Methods section.*
☒	☐	A description of all covariates tested
☒	☐	A description of any assumptions or corrections, such as tests of normality and adjustment for multiple comparisons
☐	☒	A full description of the statistical parameters including central tendency (e.g. means) or other basic estimates (e.g. regression coefficient) AND variation (e.g. standard deviation) or associated estimates of uncertainty (e.g. confidence intervals)
☐	☒	For null hypothesis testing, the test statistic (e.g. *F*, *t*, *r*) with confidence intervals, effect sizes, degrees of freedom and *P* value noted<br>*Give P values as exact values whenever suitable.*
☒	☐	For Bayesian analysis, information on the choice of priors and Markov chain Monte Carlo settings
☒	☐	For hierarchical and complex designs, identification of the appropriate level for tests and full reporting of outcomes
☒	☐	Estimates of effect sizes (e.g. Cohen's *d*, Pearson's *r*), indicating how they were calculated

Our web collection on statistics for biologists contains articles on many of the points above.

Software and code

Policy information about availability of computer code

Data collection	FACSDiva (v8.01)<br>Bruker Hystar/oTOF Control software (v3.2)<br>ZEISS ZEN (v2.0 and higher)
Data analysis	FlowJo (v10.7.1)<br>MaxQuant (v1.6.14.0)<br>FIJI (v2.0 and higher)<br>fastp (v0.20.0)<br>Salmon (v1.4.0)<br>DESeq2 (v1.30.0)<br>R (v4.0.3)<br>tximeta (v1.8.2)<br>ComplexHeatmap (v2.6.2)<br>clusterProfiler (v3.18.0)<br>ggplot2 (v3.3.2)<br>GraphPad Prism (v6.0 and higher)<br>Integrated Genome Viewer (v2.4.15)

For manuscripts utilizing custom algorithms or software that are central to the research but not yet described in published literature, software must be made available to editors and reviewers. We strongly encourage code deposition in a community repository (e.g. GitHub). See the Nature Portfolio guidelines for submitting code & software for further information.

Data

Policy information about availability of data

All manuscripts must include a data availability statement. This statement should provide the following information, where applicable:

- Accession codes, unique identifiers, or web links for publicly available datasets
- A description of any restrictions on data availability
- For clinical datasets or third party data, please ensure that the statement adheres to our policy

The RNA-seq data data generated in this study have been deposited in the ArrayExpress database under accession code E-MTAB-10301 [http://www.ebi.ac.uk/arrayexpress/experiments/E-MTAB-10301].

The proteomics data data data generated in this study have been deposited in the ProteomeXchange database under accession code PXD025293 [http://www.ebi.ac.uk/pride/archive/projects/PXD025293]

Uniprot database UP000005640_9606.fasta; version April 2019 used for Mass Spectrometry analysis is available at [ftp://ftp.uniprot.org/pub/databases/uniprot/previous_releases/release-2019_04/knowledgebase/]

There is no restriction on data availability. Source data are provided with this paper.

Field-specific reporting

Please select the one below that is the best fit for your research. If you are not sure, read the appropriate sections before making your selection.

[x] Life sciences [ ] Behavioural & social sciences [ ] Ecological, evolutionary & environmental sciences

For a reference copy of the document with all sections, see nature.com/documents/nr-reporting-summary-flat.pdf

Life sciences study design

All studies must disclose on these points even when the disclosure is negative.

Sample size	For RNA-sequencing analysis, we analyzed 3 samples for each genotype, providing sufficient sequencing depth. For assessment of blebbing rates, we analysed at least 18 cells per genotype and treatment providing information about the membrane dynamics with single cell resolution. For teratomas, we analysed at least 4 teratomas per genotype to generate sufficient material for histological analysis. For analyses of preimplantation embryo chimeras, we analysed at least 6 embryos per genotype to trace the contribution of donor cells in individual embryos. For analyses of post-implantation embryo chimeras, we examined at least 32 embryos per genotype providing sufficient material to determine the level of chimerism. For Seahorse automatic flux analyses, we analyzed at least 9 wells per genotype providing sufficient material to detect modulations in the cellular metabolism.<br>No statistical method was used to determine sample size.
Data exclusions	There was no data exclusion.
Replication	Experiments were repeated independently with similar results. The number of repetitions for each experiment is stated in the figure legends.
Randomization	Not applied because of the limited number of different conditions (e.g. genotypes - wild type vs knock out).
Blinding	Not applied as the experiments are descriptive in nature and performed by single person (most of them by the first author) who also kept track of the identity of the compared samples.

Reporting for specific materials, systems and methods

We require information from authors about some types of materials, experimental systems and methods used in many studies. Here, indicate whether each material, system or method listed is relevant to your study. If you are not sure if a list item applies to your research, read the appropriate section before selecting a response.

Materials & experimental systems

n/a	Involved in the study
	☒ Antibodies
	☒ Eukaryotic cell lines
☒	Palaeontology and archaeology
	☒ Animals and other organisms
☒	Human research participants
☒	Clinical data
☒	Dual use research of concern

Methods

n/a	Involved in the study
☒	ChIP-seq
	☒ Flow cytometry
☒	MRI-based neuroimaging

Antibodies

Antibodies used

B-actin Sigma A5316 RRID:AB_476743
AE-catenin Cell Signaling 3236S RRID:AB_10827873
AE-catenin Thermo 13-9700 RRID:AB_2533044
AN-catenin Cell Signaling CD664 N/A
B-catenin BD Biosciences 610154 RRID:AB_397555
Biotin antibody agarose ImmuneChem ICP0615 N/A
Cdx2 Biogenex MU392A-UC RRID:AB_2650531
DAPI Roth 6335.1 N/A
Hoechst 33342 Sigma 14533 N/A
E-cadherin BD Biosciences 610182 RRID:AB_397581
Eomes/Tbr2 Abcam AB23345 RRID:AB_778267
Eplin Proteintech 16639-1-AP RRID:AB_2136657
Eplin Bethyl A300-103A-M RRID:AB_2779011
Eplin home-made (Abe & Takeichi, 2008)
Esrrb R&D PP-H6705-00 RRID:AB_1964232
Gapdh Cell Signaling 5174S RRID:AB_10622025
Gata3 Cell Signaling 5852S RRID:AB_10835690
GFP R&D AF4240 RRID:AB_884445
HA-tag Cell Signaling 3724S RRID:AB_1549585
Nanog Abcam ab80892 RRID:AB_2150114
N-cadherin BD Biosciences 610920 RRID:AB_2077527
Oct4 Santa Cruz sc-5279 RRID:AB_628051
Oct4 Cell Signaling 83932S RRID:AB_2721046
Rabbit IgG HRP Linked GE Healthcare NA934 RRID:AB_772206
RFP Biomol 600-401-379 RRID:AB_2209751
Secondary Donkey anti-mouse AF 488 Invitrogen A-21202 RRID:AB_141607
Secondary Donkey anti-mouse AF 594 Invitrogen A-21203 RRID:AB_141633
Secondary Donkey anti-mouse AF 647 Invitrogen A-31571 RRID:AB_162542
Secondary Donkey anti-goat AF 488 Invitrogen A-11055 RRID:AB_2534102
Secondary Donkey anti-rabbit AF 488 Invitrogen A-21206 RRID:AB_2535792
Secondary Donkey anti-rabbit AF 594 Invitrogen A-21207 RRID:AB_141637
Secondary Donkey anti-rat AF 647 Invitrogen A21247 RRID:AB_141778
Sox2 Calbiochem 246510 N/A
Sox2 Cell Signaling 23064S RRID:AB_2714146
Sox17 R&D AF1924 RRID:AB_355060
Peroxidase AffiniPure Goat Anti-Mouse IgG + IgM (H+L) Jackson ImmunoResearch AB_2338451 RRID:AB_2338451
Phalloidin AF647 Cell Signaling 8940S N/A
Phospho-ERM Cell Signaling 3726S RRID:AB_10560513
Pierce High Sensitivity Streptavidin-HRP Thermo 21130 N/A
Troma-1 home-made (Kemler et al., 1981)

Validation

The primary antibodies in this study are previously validated and all relevant information can be found in the Resource Identification Portal using the respective RRID number.

Eukaryotic cell lines

Policy information about cell lines

Cell line source(s)

Mouse: MEF_DR-4 gift from Prof. Dr. Hans R. Schöler
Mouse: MEF_CF-1 gift from Prof. Dr. Hans R. Schöler
Mouse: ESC_WT_E14 gift from Prof. Dr. Hans R. Schöler
Mouse: ESC_WT_E14 gift from Prof. Dr. Rolf Kemler
Mouse: ESC_E14_lima1KO This paper

	Mouse: ESC_E14_lima1KO_H2B-tdTomato This paper<br>Mouse: ESC_WT_R1 129X1/SvJ x 129S1/Sv-Oca2+Tyr+KitlSl-J<br>Mouse: ESC_R1_Lima1-HA-APEX2 This paper<br>Mouse: ESC_mT/mG (Ozguldez et al., 2020)<br>Mouse: EpiSC_WT_E3 (D. W. Han et al., 2010)<br>Mouse: EpiSC_E3_Venus This paper<br>Mouse: EpiSC_E3_Lima1-HA-Venus This paper<br>Human: hiPSC_WT_C3-5 (Kim et al., 2020)<br>Human: hiPSC_C3-5_Venus This paper<br>Human: hiPSC_C3-5_Lima1-HA-Venus This paper<br>Human: WT_niPSCs (Guo et al., 2017)<br>Mouse: WT_TSCs (Kubaczka et al., 2014)
Authentication	The genotype of all newly established cell lines were verified by PCR, Western blot or immunofluorescence. The results of these analysis are provided in the main and supplementary figures.
Mycoplasma contamination	Not tested.
Commonly misidentified lines<br>(See ICLAC register)	No commonly misidentified lines.

Animals and other organisms

Policy information about studies involving animals; ARRIVE guidelines recommended for reporting animal research

Laboratory animals	Mus musculus; B6C3F1, CD1; females; 5 - 25 weeks
Wild animals	No wild animals used.
Field-collected samples	No samples colllected from the field.
Ethics oversight	Animal experiments and husbandry were performed according to the German Animal Welfare guidelines and approved by the Landesamt für Natur, Umwelt und Verbraucherschutz Nordrhein-Westfalen (State Agency for Nature, Environment and Consumer Protection of North Rhine-Westphalia)

Note that full information on the approval of the study protocol must also be provided in the manuscript.

Flow Cytometry

Plots

Confirm that:

- [x] The axis labels state the marker and fluorochrome used (e.g. CD4-FITC).
- [x] The axis scales are clearly visible. Include numbers along axes only for bottom left plot of group (a 'group' is an analysis of identical markers).
- [x] All plots are contour plots with outliers or pseudocolor plots.
- [x] A numerical value for number of cells or percentage (with statistics) is provided.

Methodology

Sample preparation	The cells were dissociated using trypsin and transferred into PBS supplemented with 3% FCS.
Instrument	FACSAria IIIu and FACSAria Fusion systems
Software	FACSDiva for sorting and FlowJo for analysis
Cell population abundance	After the sorting the cell population identity and abundance was verified by transgene expression using immunoflourescence staining.
Gating strategy	Single viable cells were first selected based on forward scatter area/side scatter area and forward scatter width/forward scatter area gating to select for live cells (DAPI negative) and then sorted for the fluorophore expression.

- [x] Tick this box to confirm that a figure exemplifying the gating strategy is provided in the Supplementary Information.
